# Cancer-associated mesothelial cell–derived ANGPTL4 and STC1 promote the early steps of ovarian cancer metastasis

**DOI:** 10.1172/jci.insight.163019

**Published:** 2023-03-22

**Authors:** Preety Bajwa, Kasjusz Kordylewicz, Agnes Bilecz, Ricardo R. Lastra, Kristen Wroblewski, Yuval Rinkevich, Ernst Lengyel, Hilary A. Kenny

**Affiliations:** 1 Department of Obstetrics and Gynecology/Section of Gynecologic Oncology,; 2 Department of Pathology, and; 3 Department of Public Health Sciences, University of Chicago, Chicago, Illinois, USA.; 4 Institute of Regenerative Biology and Medicine, Helmholtz Zentrum München, Munich, Germany.

**Keywords:** Cell Biology, Oncology, Cancer, Mouse models, Obstetrics/gynecology

## Abstract

Ovarian cancer (OvCa) preferentially metastasizes in association with mesothelial cell–lined surfaces. We sought to determine if mesothelial cells are required for OvCa metastasis and detect alterations in mesothelial cell gene expression and cytokine secretion upon interaction with OvCa cells. Using omental samples from patients with high-grade serous OvCa and mouse models with *Wt1*-driven GFP-expressing mesothelial cells, we validated the intratumoral localization of mesothelial cells during human and mouse OvCa omental metastasis. Removing mesothelial cells ex vivo from human and mouse omenta or in vivo using diphtheria toxin-mediated ablation in *Msln*-Cre mice significantly inhibited OvCa cell adhesion and colonization. Human ascites induced angiopoietin-like 4 (ANGPTL4) and stanniocalcin 1 (STC1) expression and secretion by mesothelial cells. Inhibition of STC1 or ANGPTL4 via RNAi obstructed OvCa cell-induced mesothelial cell to mesenchymal transition while inhibition of ANGPTL4 alone obstructed OvCa cell-induced mesothelial cell migration and glycolysis. Inhibition of mesothelial cell ANGPTL4 secretion via RNAi prevented mesothelial cell–induced monocyte migration, endothelial cell vessel formation, and OvCa cell adhesion, migration, and proliferation. In contrast, inhibition of mesothelial cell STC1 secretion via RNAi prevented mesothelial cell–induced endothelial cell vessel formation and OvCa cell adhesion, migration, proliferation, and invasion. Additionally, blocking ANPTL4 function with Abs reduced the ex vivo colonization of 3 different OvCa cell lines on human omental tissue explants and in vivo colonization of ID8^p53–/–Brca2–/–^ cells on mouse omenta. These findings indicate that mesothelial cells are important to the initial stages of OvCa metastasis and that the crosstalk between mesothelial cells and the tumor microenvironment promotes OvCa metastasis through the secretion of ANGPTL4.

## Graphical abstract

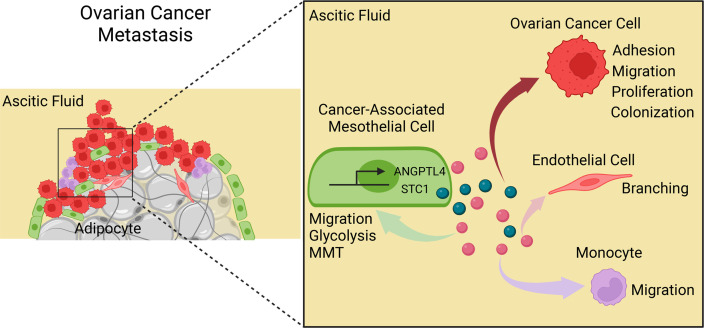

## Introduction

Ovarian cancer (OvCa) is the most lethal gynecological malignancy and the fifth leading cause of cancer-related deaths of women in the United States (1). The most prevalent and aggressive histologic subtype is high-grade serous OvCa, associated with the accumulation of malignant ascites and widespread metastases throughout the peritoneal cavity (2, 3). OvCa departs from the usual metastatic pattern of hematogenous spreading cancers. After OvCa cells detach from the primary tumor site, which can be either the ovary or fallopian tube, they are carried passively by the physiological movement of peritoneal fluid disseminating throughout the peritoneal cavity (4). They then attach to the mesothelium, which serves as the first and primary microenvironment encountered by OvCa cells in the abdominal and pleural cavity (4).

The mesothelium is an epithelial monolayer derived from the embryonic mesoderm, consisting of cobblestone-like mesothelial cells that line the coelomic cavities and internal organs (5). The mesothelium provides a frictionless interface between adjacent organs and their cavities through the synthesis of phospholipids and glycosaminoglycans. Additional functions include cell and fluid transport and secretion of proinflammatory cytokines and growth factors when encountering infection (6). During wound healing, peritoneal fibrosis, and surgical adhesion formation, mesothelial cells are reprogrammed into mesenchymal cells (6–10).

Mesothelial cells were thought to play a passive role in cancer metastasis, serving only as a barrier to be displaced by invading cancer cells (11, 12). However, more recent studies highlight the active role of mesothelial cells in promoting metastasis. In gastric cancer (13) and OvCa (14), mesothelial cells were found at the leading edge of cancer cell invasion, which could be blocked by inhibiting mesothelial cell movement. However, the direct role of mesothelial cells during the initial stages of OvCa metastasis and the global changes cancer cells and ascites fluid induce in mesothelial cells have not yet been elucidated.

In this study, we hypothesized that OvCa cells secrete factors that transform normal mesothelial cells into tumor-promoting cancer-associated mesothelial cells. To test this hypothesis, the fate of mesothelial cells during OvCa progression was traced in mice, and their tumor-promoting role was evaluated. We found that mesothelial cells are peri- and intratumoral components of OvCa tumors and that mesothelial cells are essential for OvCa metastases. Two secreted proteins in human mesothelial cells, angiopoietin-like 4 (ANGPTL4) and stanniocalcin-1 (STC1), were the genes/proteins most significantly regulated by ascites and were clearly instrumental in the creation of a protumorigenic microenvironment.

## Results

### Surface and intratumoral localization of mesothelial cells during OvCa metastasis.

**Figure 1:**
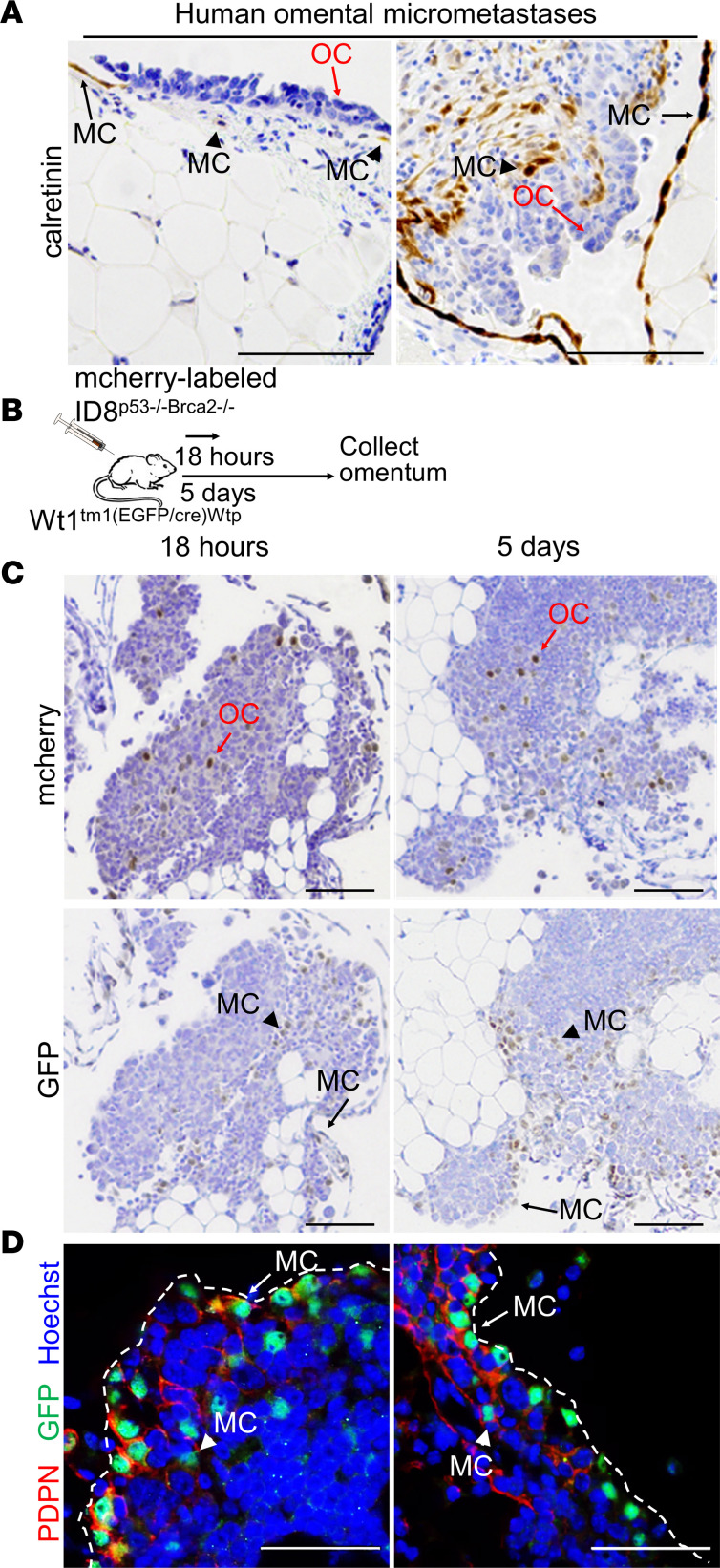
Localization of mesothelial cells during OvCa metastasis. (**A**) IHC localization of calretinin in omental samples from patients with high-grade serous ovarian cancer (OC) with micrometastases. Black arrows, mesothelial cells (MCs) located on the surface of the omentum and adjacent to OC cells. Black arrowheads, MCs located within the tumor. Red arrows, OC cells. (**B**) Mouse study design for **C** and **D** *Wt1^tm1(EGFP/Cre)Wtp^/J* heterozygous mice received an i.p. injection of mCherry-labeled ID8^p53–/–Brca2–/–^ cells and omenta was collected 18 hours and 5 days after injection. (**C**) IHC localization of mCherry or GFP in mouse omenta. Black arrows, GFP-expressing MCs located on the surface of tumor. Black arrowheadss, GFP-expressing intratumoral MCs. (**D**) Immunofluorescence. Localization of podoplanin (PDPN, red) and GFP (green) in mouse omenta. Nuclei are stained with Hoechst (blue). Dotted white line is the surface of the tumor/omentum. White arrows point to double-positive MCs on the surface. White arrowheads point to double-positive intratumoral MCs. Scale bar: 500 μm.

Calretinin, a calcium-binding protein, is expressed in mesothelial cells (15). To locate mesothelial cells during human omental metastasis (Figure 1A), IHC for calretinin was performed. Calretinin-expressing mesothelial cells were detected on the surface of the omenta in areas without tumor as well as in micrometastasis.

*Wt1^tm1(EGFP/Cre)Wtp^/J* heterozygous mice, which have GFP-expressing mesothelial cells under the control of the Wt1 promoter, were used to determine the fate of mesothelial cells during OvCa metastasis (Figure 1B and Supplemental Figure 1; supplemental material available online with this article; https://doi.org/10.1172/jci.insight.163019DS1). The omenta of the mice were collected at 18 hours and at 5 days after i.p. injection of mCherry-labeled ID8^p53–/–Brca2–/–^ mouse OvCa cells because by 18 hours, OvCa cells have adhered to and invaded the omentum and by 5 days, they have colonized it. The presence of GFP-expressing mesothelial cells was detected on the surface of the omenta in areas with and without mCherry-expressing cancer cells, as well as intratumorally (Figure 1C). Since both mesothelial cells and adipocytes express Wt1, we validated the presence of mesothelial cells in these tumors using coimmunofluorescence with Abs to GFP and to podoplanin (PDPN), a mucin-type transmembrane protein expressed in mesothelial cells (16) (Figure 1D). Following OvCa cell injection, mesothelial cells that coexpressed PDPN and GFP were observed on the surface of the tumor, as well as intratumorally. These results indicate that mesothelial cells are part of the OvCa omental tumors and are not just displaced by invading cancer cells as previously suggested (11).

### Mesothelial cells are an essential component during OvCa metastasis.

**Figure 2:**
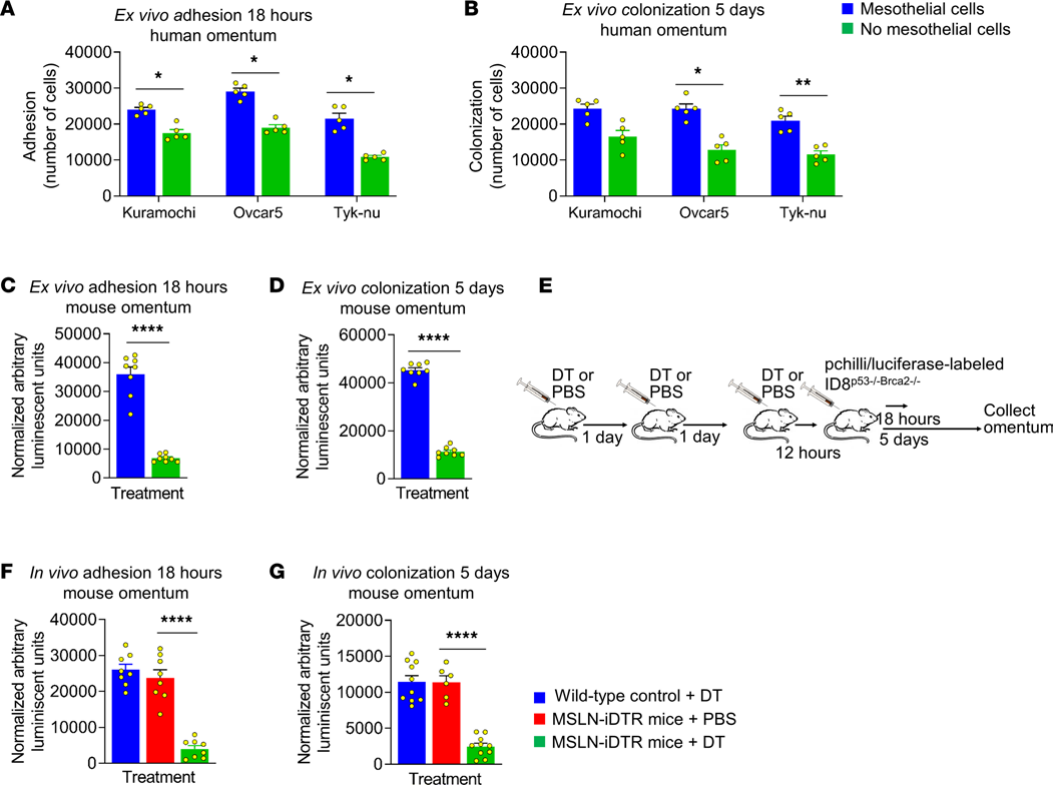
Depletion of mesothelial cells from the omentum inhibits OvCa cell adhesion and colonization. (**A** and **B**) Human ex vivo adhesion and colonization assays. Human omental tissue explants were digested with pronase before seeding of GFP-labeled Tyk-nu, OVCAR5, or Kuramochi OvCa cells, and the number of tumor cells that adhered/colonized on the omentum was quantified after 18 hours in **A** or 5 days in **B**. The cancer cells were digested off the omentum and counted using a fluorescent imaging cytometer. Data are represented as the mean ± SEM (*n* = 5). *P < 0.05 and **P < 0.01 by 1-way ANOVA. (**C** and **D**) Mouse ex vivo adhesion and colonization assays. C57Bl/6 mouse omental tissue explants were digested with pronase before seeding of GFP-labeled ID8^p53–/–Brca2–/–^ cells, mouse OvCa cells, and the number of OvCa that adhered/colonized on the omentum were quantified at 18 hours in **C** or 5 days in **D**. The cancer cells were digested off the omentum and counted using a fluorescent imaging cytometer. Data are shown as the mean ± SEM (*n* = 8). ****P < 0.0001 using paired 2-tailed Student’s *t* test comparisons. (**E**) Experimental set-up for mouse models. MSLN-iDTR or the WT litter mates (control) were injected with DT or PBS for 3 consecutive days before transplantation with pchilli/luciferase-labeled ID8^p53–/–Brca2–/–^ cells. (**F** and **G**) Mouse omental tissues were collected at 18 hours in **F** or 5 days in **G** after cancer cell injection digested, and a luciferase assay was performed. Data are represented as the mean ± SEM (*n* = 8), ****P < 0.0001 by 1-way ANOVA.

We used both ex vivo and in vivo models to evaluate the role of mesothelial cells during OvCa metastasis. In the ex vivo studies, OvCa cell activity was assessed using omental tissue with and without pronase treatment to remove mesothelial cells. After mesothelial cells were digested off pieces of human or mouse omentum with pronase (Supplemental Figure 2, A and B), GFP-labeled human or mouse OvCa cell lines were added to both digested and undigested omental pieces. Kuramochi, OVCAR5, and Tyk-nu OvCa cell adhesion (18 hours; Figure 2A) and OVCAR5 and Tyk-nu OvCa cell colonization (5 days; Figure 2B) were significantly inhibited in the omental pieces that lacked mesothelial cells when compared with intact mesothelial cell–lined omentum (Supplemental Figure 2A). Adhesion (Figure 2C) and colonization (Figure 2D) of mouse mCherry-labeled ID8^p53–/–Brca2–/–^ OvCa cells were also inhibited when mouse omenta lacked mesothelial cells (Supplemental Figure 2B).

A transgenic mouse model was developed by crossing mesothelin-targeted (MSLN^CL^) mice (17) with C57BL/6-Gt(ROSA)26Sort^m1(HBEGF)Awai^/J mice to determine the role of mesothelial cells during OvCa metastasis in vivo (Supplemental Figure 3A). In this transgenic mouse model, Msln-, LacZ-, Cre-, and diphtheria toxin receptor-expressing (DTR-expressing) mesothelial cells were ablated with DT i.p. treatment before i.p. injection of pchili/luciferase-labeled ID8^p53–/–Brca2–/–^ mouse OvCa cells (Figure 2E and Supplemental Figure 3, A–C). Controls included transgenic mice given i.p. injections of PBS before cancer cell injection to measure any effects of the genetic background and Cre expression on OvCa metastasis, and WT litter mates injected with DT before cancer cell injection, to evaluate any effects of DT on OvCa metastasis. Adhesion (Figure 2F and Supplemental Figure 4A) and colonization (Figure 2G and Supplemental Figure 4B) of the mouse OvCa cells to the mouse omentum were significantly inhibited in the mice without mesothelial cells when compared with the 2 control groups.

### OvCa cells induce secretome and transcriptome changes in mesothelial cells.

**Figure 3:**
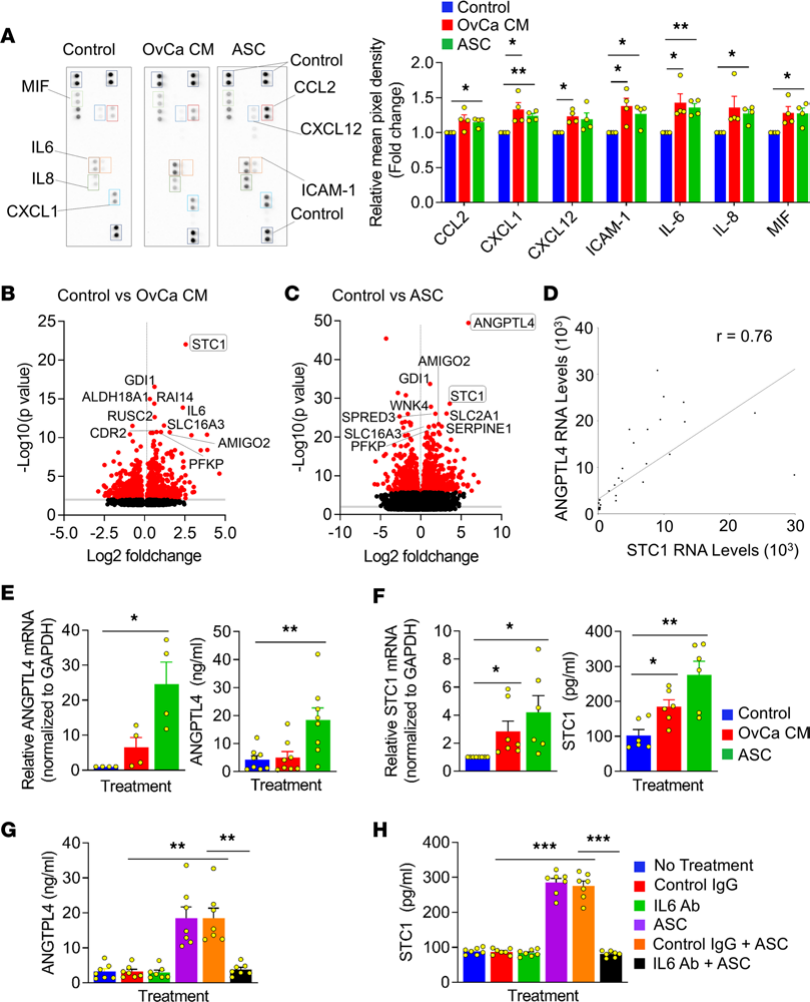
OvCa cells induce secretome and transcriptome changes in primary human mesothelial cells. (**A**) Mesothelial cells were treated with control media (Control), Tyk-nu OvCa CM, or human ascites (ASC) for 36 hours. The mesothelial cells were washed, and serum-free media was added for 24 hours. Human Cytokine Arrays were performed. One of the 4 biological replicates of each treatment are shown (left). Quantification using ImageJ software (right). Data are shown as the mean ± SEM. *P < 0.05 and **P < 0.01 by paired Student’s *t* test comparisons. (**B–D**) Mesothelial cells were treated with Control, OvCa CM, or ASC from 3 different patients (*n* = 10). RNA was isolated from the mesothelial cells and RNA-Seq analysis was performed. Volcano plots annotated with top 10 significantly upregulated transcripts in the OvCa CM in **B** or ASC-treated mesothelial cells in **C**. (**D**) Pearson’s correlation coefficient analysis of ANGPTL4 and STC1 RNA levels in control versus ascites-treated mesothelial cells from this experiment. (**E** and **F**) Mesothelial cells were treated with control media (Control), OvCa CM, or ASC. RNA was isolated from the mesothelial cells, or mesothelial cell CM was collected. ANGPTL4 in **E** and STC1 expression and secretion in **F** was quantified using reverse transcription-qPCR (qRT-PCR) (*n* = 7 patients in OvCa CM group and *n* = 6 patients in ASC group) or enzyme-linked immunostaining assays. Data are represented as the mean ± SEM (*n* = 6). *P < 0.05 and **P < 0.01 calculated using paired 2-tailed Student’s *t* test comparisons. (**G** and **H**) Mesothelial cells were treated with an IL-6–neutralizing Ab or control Ab (Control IgG) in the presence of ASC. Mesothelial cells were washed, serum-free media was added, and the mesothelial cell CM was collected. Analysis of ANGPTL4 in **G** and STC1 secretion in **H** using ANGPTL4- or STC1-specific enzyme linked immunostaining assays. Data are shown as the mean ± SEM (*n* = 7). **P < 0.01 and ***P < 0.001 by 1-way ANOVA.

To evaluate OvCa-induced changes in primary human mesothelial cell cytokine secretion, mesothelial cells were treated with OvCa cell conditioned media (CM), ascites from patients with high-grade serous OvCa or control media. The CM induced CXCL1, IL-6, ICAM-1, and CXCL12 secretion, while malignant ascites induced CXCL1, IL-6, ICAM-1, MIF, CCL2, and IL-8 secretion (Figure 3A). To evaluate the OvCa-induced changes in gene expression, primary human mesothelial cells from 10 patients were treated with OvCa CM or ascites and RNA-Seq analysis performed (Supplemental Figure 5, A–C). The top 10 upregulated genes in mesothelial cells (Figure 3, B and C) revealed that both CM and ascites treatment induced the STC1 gene in the mesothelial cells (Supplemental Table 1), while the ANGPTL4 gene was induced by ascites only (Supplemental Table 2). Additionally, both treatments induced glycolysis, epithelial-to-mesenchymal (EMT), angiogenesis, and hypoxia-related hallmark pathways (Supplemental Figure 5C). The expression of STC1 and ANGPTL4 RNA levels were positively correlated in the mesothelial cells (Figure 3D). Downregulation of 3-hydroxy-3-methylglutaryl-CoA reductase and insulin induced gene 1 expression by human ascites in mesothelial cells was validated using specific taqman gene expression assays (Supplemental Figure 5, D and E). We confirmed that ANGPTL4 expression and secretion by mesothelial cells is only induced by ascites (Figure 3E) and that STC1 expression and secretion by mesothelial cells is induced by OvCa CM and human ascites (Figure 3F). Because IL-6 was induced in mesothelial cells by ascites and CM (Figure 3A), the role of IL-6 in ascites-induced expression of ANGPTL4 and STC1 was explored. The addition of an IL-6–neutralizing Ab blocked ascites-induced ANGPTL4 (Figure 3G) and STC1 (Figure 3H) secretion in mesothelial cells. These data show that the secreted proteins ANGPTL4 and STC1 are involved in the OvCa-induced changes in primary human mesothelial cells.

### ANGPTL4 and STC1 regulate mesothelial cell functions.

**Figure 4:**
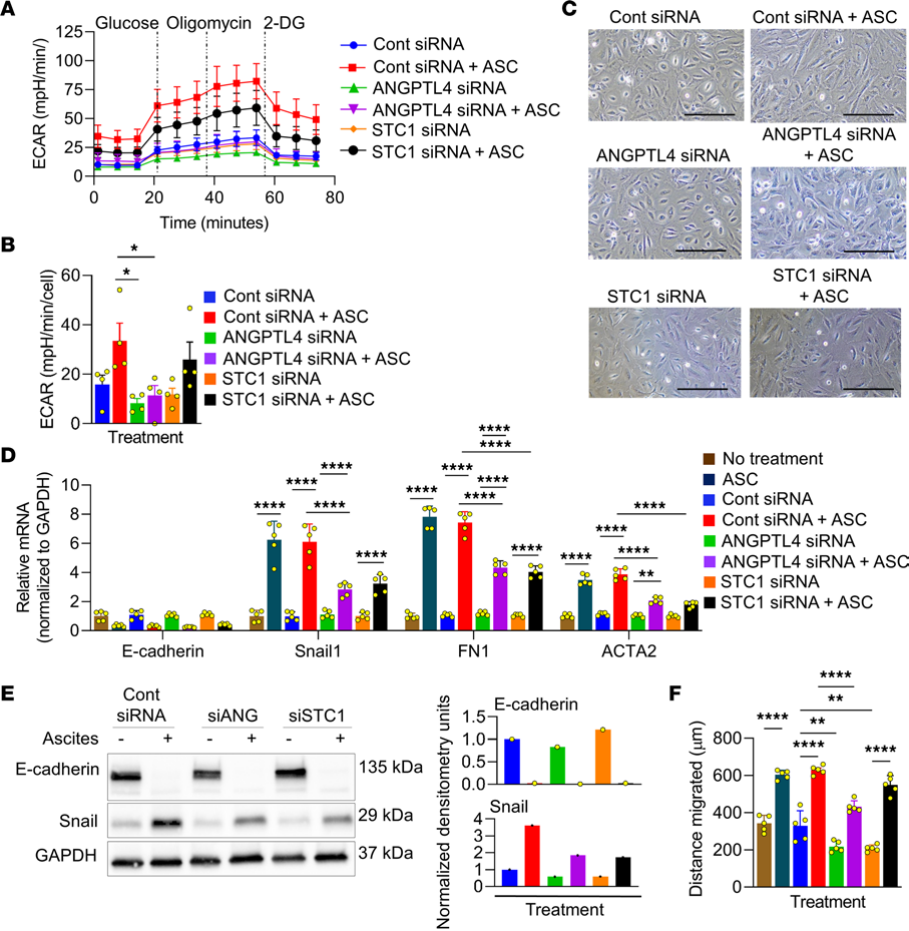
Ascites-induced glycolysis, EMT, and wound healing in mesothelial cells are regulated by ANGPTL4 and/or STC-1. (**A** and **B**) Glucose metabolism in mesothelial cells. Mesothelial cells were transfected with control, ANGPTL4, or STC1 siRNA followed by treatment with ascites (ASC) or control media, and a glycolysis stress assay performed. (**A**) Seahorse ECAR profile of mesothelial cells following glucose, oligomycin, and 2-deoxy-D-glucose treatments. (**B**) Changes in basal ECAR, glycolytic capacity, and glycolytic reserve in mesothelial cells. Data are represented as the mean ± SEM (*n* = 4). *P < 0.05 by 1-way ANOVA. (**C–E**) Mesothelial cells were transfected with control (Cont), ANGPTL4, or STC1 siRNA followed by treatment with ASC or control media. Phase-contrast images in **C**. Scale bar: 100 μm. qRT-PCR for epithelial (E-cadherin) and mesenchymal (Snail, fibronectin, and α-SMA) markers in **D**. Data are shown as the mean ± SEM (*n* = 5). **P < 0.01 and ****P < 0.0001 calculated using 2-way ANOVA. IB analysis of E-cadherin and Snail in **E**. *n* = 3 patients/group. (**F**) Wound-healing assay. Mesothelial cells were transfected with control, ANGPTL4, or STC1 siRNA, the mesothelial cell layer disrupted and treated with ASC or control media, and the distance mesothelial cell migrated was measured. Data are represented as the mean ± SEM (*n* = 5). **P < 0.01 and ****P < 0.0001 by 1-way ANOVA.

Based on the gene set enrichment analysis (Supplemental Figure 5C), we explored the roles of ANGPTL4 and STC1 in glycolysis and EMT using siRNA. The knockdown of ANGPTL4 or STC1 in the mesothelial cells inhibited ANGPTL4 and STC1 expression and secretion (Supplemental Figure 6, A and B). Knockdown of ANGPTL4, but not treatment with an ANGPTL4-neutralizing Ab, reduced glycolysis, glycolytic capacity, and glycolytic reserve in primary human mesothelial cells treated with ascites (Figure 4, A and B; and Supplemental Figure 7, A and B). Conversely, knockdown or neutralization of STC1 had little to no effect on mesothelial cell glycolysis or glycolytic capacity.

Knockdown of ANGPTL4 or STC1 inhibited ascites-induced mesenchymal morphology (Figure 4C) and classic mesenchymal markers in mesothelial cells (snail, fibronectin, and smooth muscle actin; Figure 4, D and E). Consistent with these results, the neutralization of ANGPTL4 inhibited the expression of ascites-induced snail and fibronectin (Supplemental Figure 7C). However, knockdown of ANGPTL4 and STC1 had neither effect on the ascites-induced reduction in the expression of E-cadherin (Figure 4, D and E) nor on ascites-induced hypoxia in mesothelial cells (Supplemental Figure 8). Next, we explored the role of ANGPTL4 and STC1 in the ascites-induced migration of mesothelial cells. The knockdown of ANGPTL4, but not STC1, blocked ascites-induced mesothelial cell migration (Figure 4F). Taken together, these data reveal that: (a) both ANGPTL4 and STC1 play a role in mesothelial cell mesenchymal transition and (b) ANGPTL4 regulates ascites-induced glycolysis and mesothelial cell migration.

### Mesothelial cell ANGPTL4 and STC1 expression regulates endothelial, monocyte, and OvCa cell functions.

**Figure 5:**
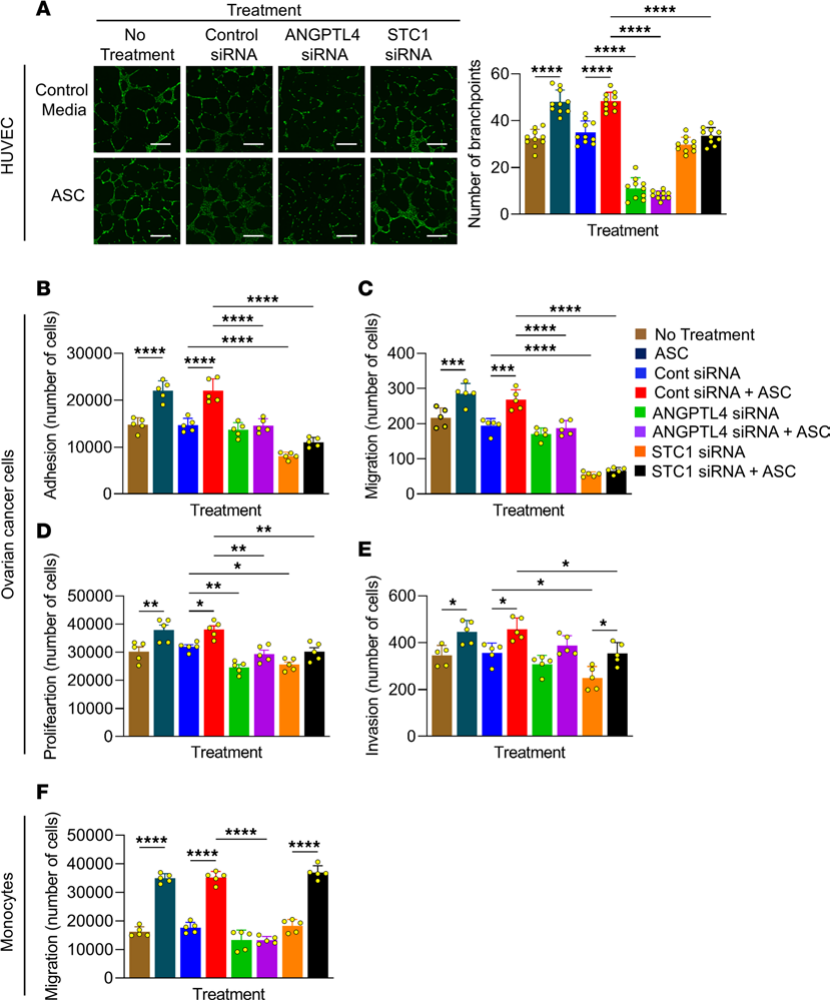
Mesothelial cell–derived ANGTPLT4 and STC1 regulate endothelial cell, monocyte, and OvCa cell function. Mesothelial cells were transfected with control, ANGPTL4, or STC1 siRNA followed by treatment with ascites (ASC) or control media. Subsequently, mesothelial cell CM was collected. (**A**) HUVECs were plated on Geltrex Matrix and treated with mesothelial cell CM. Tube formation was quantified using Calcein AM staining. Images of HUVECs and corresponding quantification. Scale bar: 300 μm. (**B–E**) Functional assays were performed using fluorescently labeled Tyk-Nu OvCa cells. OvCa cell adhesion (1 hour) was tested on top of the transfected and pretreated mesothelial cell monolayer in **B**. OvCa cell migration (12–24 hours) was tested using a Boyden chamber lined with transfected mesothelial cells in **C**. OvCa cell proliferation (72 hours) was tested on top of the transfected and pretreated mesothelial cell monolayer in **D**. OvCa cell invasion (24–48 hours) was tested through transfected and pretreated mesothelial cell monolayer and rat tail collagen type I using a Boyden chamber in **E**. (**F**) Human CD14^+^ cells were isolated from peripheral blood and placed in serum-free media in the top well of a 5 μm pore-size transwell. Mesothelial cell CM were placed in the bottom chamber and the number of cells migrated to the bottom well were recovered and counted after 4 hours. All data are shown as the mean ± SEM (*n* = 5–8). *P < 0.05, **P < 0.01, ***P < 0.001, and ****P < 0.0001 calculated by 1-way ANOVA.

Based on the gene set enrichment analysis (Supplemental Figure 5C), we tested how ANGPTL4 and STC1 regulated mesothelial cell–induced angiogenesis and found that the knockdown of ANGPTL4 or STC1 in mesothelial cells inhibited the ascites treatment-driven increase in human umbilical cord endothelial cell branch points (Figure 5A). The secreted proteins, ANGPTL4 (18, 19) and STC1 (20–22), play a role in cellular adhesion, migration, invasion, and proliferation. We found that knockdown of either ANGPTL4 or STC1 in mesothelial cells pretreated with ascites decreased the adhesion, migration, and proliferation of 3 different OvCa cells (Figure 5, B–D; and Supplemental Figure 9, A–C). However, only STC1 knockdown in mesothelial cells pre-treated with ascites decreased OvCa cell invasion through the mesothelial cell layer (Figure 5E and Supplemental Figure 9D). In contrast, knockdown of ANGPTL4, but not STC1, in mesothelial cells blocked the ascites-induced monocyte cell migration toward mesothelial cell CM (Figure 5F).

### STC1 and ANGPTL4 inhibition blocks OvCa metastasis.

**Figure 6:**
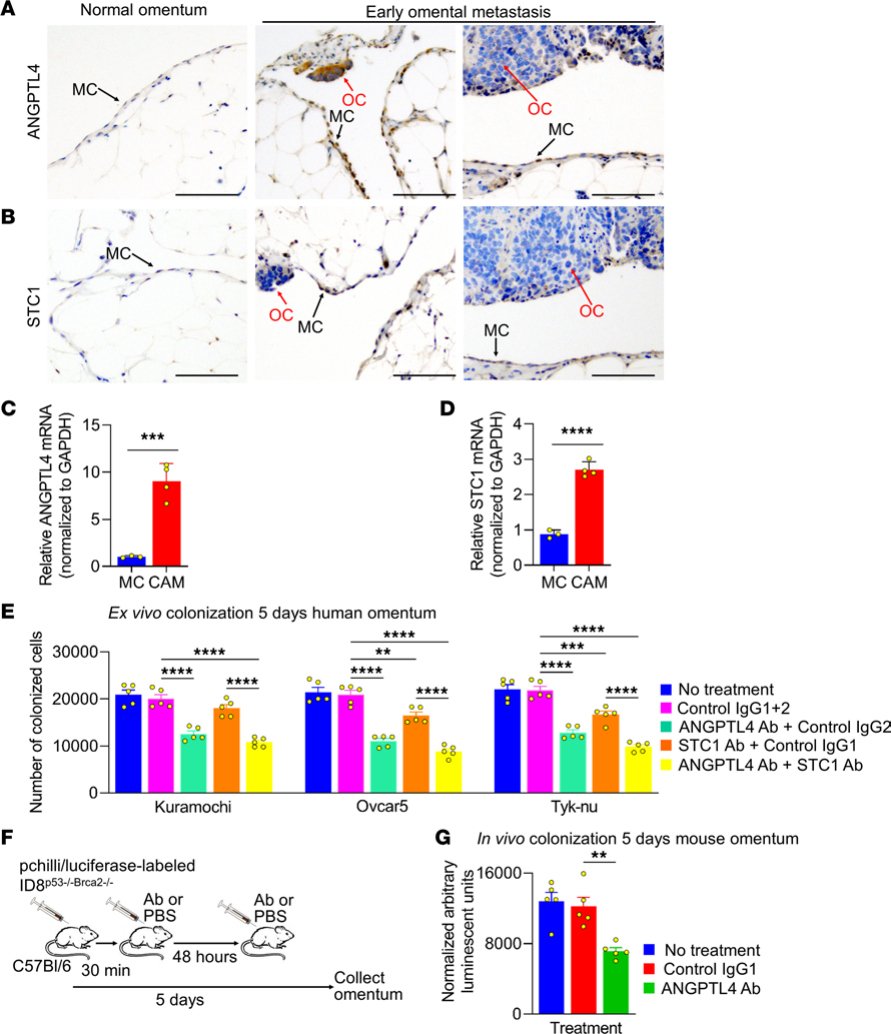
Treatment with ANGPTL4- or STC1-neutralizing Abs inhibits ovarian metastasis. (**A** and **B**) IHC localization of ANGPTL4 or STC1 in omental samples from a patient with benign disease or patients with high-grade ovarian cancer (OC) with micro-metastases. Black arrows point to mesothelial cells (MCs). Red arrows point to OC. Scale bar: 500 μm. (**C** and **D**) ANGPTL4 or STC1 mRNA expression levels in MCs or cancer-associated mesothelial cells (CAMs). HBME-1 positive cells were FACS from benign omentum washes or ascites from patients with high-grade serous OC. Data are shown as the mean ± SEM (*n* = 3–4). ***P < 0.001 and ****P < 0.0001 using unpaired Student’s *t* tests. (**E**) Human ex vivo colonization assay. GFP-labeled Tyk-nu, OVCAR5, or Kuramachi OC cells were seeded on human omental tissue explants. The cultures were untreated or treated with an ANGPTL4 neutralizing Ab, a STC1-neutralizing Ab, the respective control IgGs, or both neutralizing Abs. The cancer cells were digested off the omentum and counted using a fluorescent imaging cytometer. Data are represented as the mean ± SEM (*n* = 5). **P < 0.01, ***P < 0.001, and ****P < 0.0001 calculated by 1-way ANOVA. (**F**) Mouse study design for **G**. (**G**) C57Bl/6 mice were treated with a control IgG or ANGPTL4 Ab (5mg/kg) for 30 minutes; and 48 hours after pchili/luciferase-labeled OC cell i.p. injection, the mouse omental tissues were collected, digested, and a luciferase assay was performed. Data are shown as the mean ± SEM (*n* = 5). **P < 0.01 by 1-way ANOVA.

In normal human omental tissue, ANGPTL4 and STC1 are both expressed in mesothelial cells; however both proteins are more strongly expressed in mesothelial cells localized near OvCa cells in omental metastases (Figure 6, A and B). This was paralleled by high mRNA levels of ANGPTL4 and STC1. Primary human mesothelial isolated by FACS from the omental tissue of patients with high-grade serous OvCa had much higher ANGPTL4 and STC1 mRNA levels compared with mesothelial cells from patients with benign disease (Figure 6, C and D).

We then utilized human ex vivo and mouse in vivo models to examine the functional role of ANGPTL4 and STC1 during OvCa metastasis (Figure 6, E–G). First, we measured the effect of ANGPTL4- and STC1-neutralizing Abs on GFP-labeled OvCa cell colonization on pieces of human omentum (Figure 6E). The ANGPTL4 Ab inhibited Kuramochi (36%), OVCAR5 (47%), and Tyk-nu (41%) OvCa cell colonization on the human omentum, while the STC1 Ab inhibited only the Tyk-nu (24%) OvCa cell line. No additive or synergistic effect on OvCa colonization was observed with combinatorial treatment. Second, we investigated whether neutralization of ANGPTL4 or STC1 inhibited OvCa-induced EMT. Neutralization of ANGPTL4 inhibited the OvCa cell-induced fibronectin expression in mesothelial cells (Supplemental Figure 10). Next, an in vivo experiment with a mouse ANGPTL4-neutralizing Ab (Figure 6F) was performed to validate our ex vivo results. The i.p. injection of luciferase-labeled ID8^p53–/–Brca2–/–^ mouse OvCa cells followed by treatment with the ANGPTL4-neutralizing Ab blocked omental metastasis (Figure 6G). Taken together, these results reveal the therapeutic potential of neutralizing ANGPTL4 during OvCa metastasis to the omentum.

## Discussion

The tumor microenvironment is a heterogenous collection of cancer, resident, and infiltrating cells, as well as secreted factors and extracellular matrices (23, 24). For high-grade serous OvCa and other abdominally metastasizing tumors, the primary site of metastasis is the mesothelium, a monolayer of mesothelial cells resting on the basement membrane which lines the organs of the peritoneal and pleural cavities (4). Previously, it was thought that the mesothelial cells were a passive barrier which cancer cells displaced to get access to the submesothelial basement membrane (11). However, the experiments presented here support a more nuanced view of the role of mesothelial cells in cancer: First, we detected mesothelial cells in the omental tumor microenvironment. Second, by removing mesothelial cells ex vivo from the mouse or human omentum, or in vivo using DT-mediated mesothelial cell ablation, we prevented OvCa adhesion and colonization on the omentum. These results, in conjunction with previous studies, give evidence that, in the presence of OvCa, mesothelial cells become cancer associated and are an important component of the omental tumor microenvironment (7, 25–35). Although we did not study additional sites of peritoneal or pleural metastasis, these organs, like the omentum, are lined by a single layer of mesothelial cells (6, 9). It is likely that mesothelial cells have an essential role during the metastasis of all peritoneal/pleura cavity metastasizing cancers (i.e., gastric and pancreatic cancer).

The mesothelium has several physiologic functions, including transport and movement of fluid and particulate matter, and synthesis of proinflammatory cytokines, growth factors, and extracellular matrices during serosal repair (6, 10, 36). Mesothelial cells become more mesenchymal during wound healing, peritoneal fibrosis, surgical adhesion formation, and dialysis (6, 7, 9, 10). We performed a systematic analysis of changes induced in mesothelial cells within the OvCa tumor microenvironment as well as their functional implications by defining the secretome and transcriptome changes in primary human omental mesothelial cells. OvCa cells induced major changes in mesothelial cells, including glycolysis, angiogenesis, hypoxia, and EMT, in a manner consistent with a previous report that ascites induce cell-to-cell signaling in mesothelial cells (37). In addition, we found that cancer-associated mesothelial cells induced monocyte migration and promoted OvCa cell adhesion, migration, invasion, and proliferation. Our report establishes that mesothelial cells are a critical component of the tumor organ and help cancer cells grow and metastasize. Our data also suggest that the surgical adhesions and postoperative infections that activate mesothelial cells in the peritoneal cavity (5, 9, 10, 38) may promote OvCa metastasis.

ANGPTL4 and STC1 were among the top candidate genes that were significantly regulated by ascites in mesothelial cells. ANGPTL4 is a multifaceted protein that functions in the tumor microenvironment to promote tumor growth, angiogenesis, and chemoresistance (39–43). ANGPTL4 is secreted by multiple cell types in the tumor microenvironment, including adipocytes and cancer-associated fibroblasts, and is associated with a poor prognosis in breast, gallbladder, and OvCas (19, 41, 44–46). Furthermore, ANGPTL4 has been implicated in carboplatin resistance and hypoxia-induced anoikis resistance (40, 41). STC1 is a glycoprotein that functions as a paracrine/autocrine factor involved in many physiological/pathological pathways that promote cancer cell viability, migration, proliferation, invasion, and chemoresistance (47–49). STC1 is also secreted by multiple cell types in the tumor microenvironment, including cancer cells, cancer-associated fibroblasts, macrophages, and adipocytes (48, 50–52). STC1 reversely correlates with checkpoint therapy efficacy in patients with melanoma and lung cancer and is associated with poor patient survival across multiple cancer types (49, 53–60). Using RNAi technology and neutralizing Abs, we discovered that ANGPTL4 and STC1 differentially regulated cancer-associated mesothelial cell tumor-promoting functions, and that neutralization of ANGPTL4 alone or in combination with STC1 prevented metastasis. However, more studies are needed to determine if ANGPTL4 and/or STC1 are the major drivers of tumor-promoting functions of cancer-associated mesothelial cells, and if these proteins play a key role in the chemoresistance and immunotherapy resistance of OvCa (61, 62).

In summary, we show that mesothelial cells are required for OvCa metastasis, and that cancer-associated mesothelial cells are present in the tumor microenvironment and promote OvCa metastasis. Cancer-associated mesothelial cells secrete ANGPTL4 and STC1, which directly drive OvCa, endothelial, and monocyte cell function in the tumor microenvironment, and targeting ANGPTL4 and STC1 interferes with the tumor-promoting capabilities of cancer-associated mesothelial cells. We believe the results presented here reveal the potential therapeutic benefits of targeting mesothelial cell function during OvCa metastasis.

## Methods

### Cell lines and reagents.

All resources for these studies are described in Supplemental Tables 3 and 4. All cell lines used in this paper are listed in Supplemental Table 3. Tyk-nu, OVCAR5, and Kuramochi were cultured in DMEM with 10% FB-Essence, 1% penicillin/streptomycin, 1% MEM nonessential aa, and 1% MEM vitamins. ID8p53^–/–^Brca2^–/–^ mouse OvCa cells were maintained in DMEM supplemented with 4% FBS, Insulin-Transferrin-Selenium X, 1% penicillin/streptomycin, 1% MEM nonessential aa, and 1% MEM vitamins. All cell lines were tested every 4 months for mycoplasma using the STAT-Myco Kit and validated using short tandem repeat DNA fingerprinting with the AmpFISTR Identifier Kit and compared with known fingerprints by IDEXX BioAnalytics Laboratories.

### Mouse strains.

*Wt1^GFPCre^* (JAX stock 010911) *and* *Gt (ROSA)^26Sorttm1(HBEGF)Awai^* (JAX stock 007900), commonly known as Rosa26iDTR mice, were obtained from the Jackson Laboratory. *Msln^CLN^* transgenic mice were previously described (17). The following primers were used for genotyping: *MslnCLN*, 5′ GGAGAAAGCAAGCTCCCAACTCATGA 3′, 5′ CCACTGCTGTGTTCCAGAAGTGTTGGT 3′, and 5′ GGGACAAGTGGGGACCTCAGAGTCA 3′; and *Wt1GFPCre*, 5′ CACTACCAGCAGAACACCCCCATC 3′, 5′ TTGCGAACCTCATCACTCGTTGC 3′, 5′ GGCTTAAAGGCTAACCTGGTGTG 3′, and 5′ GGAGCGGGAGAAATGGATATG 3′.

To establish the MSLN-iDTR mouse model, *Msln*^CLN^ mice were crossed with Rosa26iDTR mice. All mice were genotyped. Littermates were assigned randomly to the experimental groups. Animals were housed under specific pathogen-free conditions with ad libitum access to food and water. All procedures involving animal care were approved by the IACUC at the University of Chicago.

### Primary human tissue collection and primary cell culture.

Specimens of fresh human omentum were obtained from consenting patients undergoing surgery for benign conditions. Specimens of fresh human omental metastases and malignant ascites were obtained from consenting patients undergoing surgery for OvCa at The University of Chicago, Section of Gynecologic Oncology. The protocols were approved by the University of Chicago Institutional Review Board. Primary human mesothelial cells were isolated from normal human omentum as previously described and were used within 2 passages of isolation (12, 63). Primary human monocytes were isolated from human peripheral blood using positive immunomagnetic separation with CD14 microbeads (Miltenyi Biotec) following the manufacturers’ protocol.

### Fluorescence-activated cell sorting of mesothelial cells.

Positive selection of mesothelial cell antibody clone (HBME-1)–stained mesothelial cells was performed as previously described (64). Briefly, primary mesothelial cells were isolated from normal human omentum and cancer-associated mesothelial cells were isolated from malignant ascites derived from patients with high-grade serous OvCa. The isolated cells were pelleted, washed with 1% BSA in PBS, and incubated in 1% BSA PBS with HBME-1 Ab (1:50). After 30 minutes of treatment, the cells were washed twice with 1% BSA PBS and incubated in 1% BSA PBS with Alexa Fluor 594 (1:1,000). After a 30-minute incubation, the cells were washed twice with 1% BSA PBS and sorted using a FACS Aria III fusion cell sorter (BD Biosciences).

### siRNA transfections.

Human primary mesothelial cells were transfected with STC1 siRNA (25 nm), ANGPTL-4 siRNA (25 nm), or negative control siRNA (20 nm) using Lipofectamine 2,000 for 24 hours, followed by treatment with control media, Tyk-nu CM, or human ascites. For the IL-6 blocking studies, an IL-6–neutralizing Ab or control IgG (3μg/mL) was added with the malignant ascites. Forty-eight hours after treatment, the cells were collected for mRNA and protein isolation.

### Generation of CM and cell treatments.

For the generation of OvCa spheroid CM, 3 × 10^6^ Tyk-nu cells were grown in ultralow attachment plates (75 cm^2^) for 72 hours at 5% CO_2_, 37°C. The cell supernatants were collected after centrifugation, filtered through a 0.45 μm filter, and immediately used or stored at –80°C for later use. For the generation of primary human mesothelial cell CM, cells were seeded at 4 × 10^5^ cells in 6-well tissue culture plates and incubated at 37°C until cuboidal morphology was observed and 80% cell confluence was reached. The mesothelial cells were then treated with control media, Tyk-nu CM, or human ascites. After 36 hours, the media was changed to serum-free media, and the cells were incubated at 5% CO_2_, 37°C. After 24 hours, the media was collected, centrifuged at 0.5*g* for 5 minutes, filtered through a 0.22 μm filter, and immediately used for endothelial and cancer cell functional assays.

### In vivo mesothelial cell tracing.

For in vivo tracing of mesothelial cells, *Wt1GFPCre* mice were injected i.p. with 5 × 10^6^ ID8p53^−/−^Brca2^–/–^ cells and omenta harvested at 18 hours (for initial invasion) and 5 days (for colonization) after the injection. Immunofluorescence was performed on tissue as described below for formalin fixed tissue.

### In vivo adhesion and colonization assays.

For the studies in Figure 2F, heterozygous MSLN-iDTR mice (expressing the Msln, Cre, LacZ, and DTR) or WT littermates were randomized into experimental groups (*n* = 8–10 mice/group). A tamoxifen diet (400mg/kg; Envigo) was provided ad libitum for 14 days. On day 15, all mice received a regular chow diet, and the mice received i.p. injections of DT (100 ng/100 μL) or solvent control (PBS) every 24 hours for 3 days. On the third day, the mice were injected i.p. with 5 × 10^6^ ID8^p53–/–Brca2–/–^ pchili/luciferase cells in cold PBS (500 μL total).

For the treatment studies in Figure 6G, C57BL/6 mice were injected i.p. with 5 × 10^6^ ID8^p53–/–Brca2–/–^ pchili/luciferase-labeled cells in cold PBS (500 μL total) and randomly split up into the no treatment, control IgG, or ANGTPL4 Ab treatment groups (*n* = 5). Each mouse group received either an i.p. injection of PBS, control IgG, or ANGPTL4 Ab (5mg/kg) 30 minutes and 48 hours following cancer cell injection. The mice were sacrificed 16 hours or 5 days following cancer cell injection. The omentum was removed, omental lysates were prepared in 1X reporter lysis buffer, and total luminescence was measured using a luminometer. The luminescence was detected using a Luciferase Assay System (Promega) and normalized to total protein in the lysate as described previously (65).

### Mouse tissue IHC and immunofluorescence.

Harvested mouse omental tumors were formalin fixed prior to paraffin embedding. The fixed tissues were dehydrated using increasing dilutions of ethanol, cleared in xylene, and embedded in paraffin wax and 5 μm thick sections were mounted on Superfrost Plus charged slides (Thermo Fisher Scientific). The slides were deparaffinized in xylene and rehydrated in decreasing dilutions of ethanol. Antigen retrieval was performed in 10 mmol/L sodium citrate buffer (0.05% Tween-20, pH 6) for 30 minutes at 100°C followed by incubation with endogenous peroxide block using 3% (v/v) hydrogen peroxide in absolute methanol for 20 minutes at room temperature. The slides were blocked in 10% normal goat serum in PBS, 0.1% Triton-X 100, and incubated overnight at 4°C with primary Abs against mCherry (1:300) and GFP (1:100). The staining was visualized using the ready to use VECTASTAIN Elite ABC-HRP Kit and DAB Substrate Kit and counterstained with hematoxylin. Images were photographed using the Nikon Eclipse Ti2 microscope.

For immunofluorescence, all slides were blocked in 10% normal goat serum in PBS, 0.1% Triton-X 100 after antigen retrieval step, and incubated overnight at 4°C with primary Abs against PDPN (1:100), CK-19 (1:100), and/or GFP (1:50), followed by incubation with Alexa Fluor 488- or Alexa Fluor 568-labeled secondary Abs (1:250). Nuclei were stained with Hoechst 33342 nucleic acid stain (1:1,000 in 1% BSA in PBST) and slides were washed and mounted with ProLong Gold Antifade. Negative controls for the immunostaining were prepared by omitting the primary Ab. Images were photographed using the live cell DSU Spinning Disk Confocal microscope (Olympus).

### Human tissue IHC.

Harvested human omental tumors were formalin fixed prior to paraffin embedding. The fixed tissues were dehydrated using increasing dilutions of ethanol, cleared in xylene, and embedded in paraffin wax. Sections 5 μm thick were mounted on Superfrost Plus charged slides (Thermo Fisher Scientific). The slides were deparaffinized in xylene and rehydrated in decreasing dilutions of ethanol. Antigen retrieval was performed in epitope retrieval solution I (Leica Biosystems, AR9961) for 20 minutes. The slides were stained for 60 minutes using the Leica Bond RX automated research stainer with primary Abs anti-Calretinin (1:600), anti-ANGPL4 (1:100), or anti-STC1 (1:800). The antigen-Ab binding was detected with bond polymer refine detection (Leica Biosystems, DS9800). The primary Ab was omitted in the negative controls. Images were photographed using the Nikon Eclipse Ti2 microscope.

### IBs.

Cells were lysed in ice-cold radioimmunoprecipitation assay buffer (RIPA, 100 mM Tris/HCl, pH 7.4) supplemented with phosphatase and protease inhibitors. Equal amounts of protein (30 μg) were loaded and separated using a 4%–20% SDS-PAGE gel and then transferred to a nitrocellulose membrane. The membranes were blocked in 5% nonfat dry milk in Tris-buffered saline with 0.1% Tween-20 (TBST) for 1 hour at room temperature and probed overnight with primary Abs against E-cadherin, Snail, and β-actin (1:1,000 in 5% BSA in TBST) at 4°C. The membranes were washed 3 times with TBST and incubated with HRP-conjugated anti-mouse or anti-rabbit secondary Abs at 1:2,000 dilution in 5% nonfat dry milk in TBST for 1 hour at room temperature. Protein bands were visualized using Clarity Western ECL Substrate (Bio-Rad) using the ChemiDoc XRS+ system (Bio-Rad).

### Cytokine array.

Primary human mesothelial cells were seeded at 2.5 × 10^6^ cells per 10 cm diameter culture dish and incubated at 5% CO_2_, 37°C, until cuboidal morphology and cell confluence was reached. The cells were treated with Tyk-nu spheroid CM, malignant ascites, or control medium for 36 hours. The cells were washed and incubated with serum free media for an additional 24 hours. This mesothelial cell CM was collected, filtered through a 0.22 μm filter, and processed with the Proteome Profiler Human Cytokine Panel Array Kit (R&D Systems) following the manufacturer’s recommendations. Images were acquired on a G:BOX Chemi XT4 imager and quantified using ImageJ (NIH).

### Wound-healing mesothelial cell migration assay.

In a 96-well tissue plate, 10,000 primary human mesothelial cells and 20,000 GFP-labeled OvCar5, Tyk-nu, or Kuramochi cells were seeded per well (100 μL/well) in mesothelial cell culture media. The cells were incubated at 37°C until they reached 100% confluence (24 hours). Media was then removed, and each well was washed twice with sterile PBS. After washing, wounds were made using the 96-pin IncuCyte WoundMaker (Essen BioScience) following the provided protocol. The quality of the wounds was checked under a light microscope and after a successful quality control, the wells were replenished with 100 μL of fresh cell culture media. The cell plate was then placed into the IncuCyte S2 Live-Cell Analysis System (Sartorius) and images were taken at consistent time intervals with a 4× objective until the wound closed. The width of the wound was measured using ImageJ.

### Tube formation assay.

Twenty-four well plates were coated with 100 μL/well of Geltrex Matrix. A total of 50,000 human umbilical vein endothelial cells were plated/well on top of the matrix-coated wells in LVES-supplemented Medium 200. After 2 hours, the media was removed, and the cells were treated with fresh mesothelial cell CM (described above) or control growth media. After 12 hours, the cells were stained with 2 μg/mL of Calcein AM, and images were captured using a Nikon Eclipse Ti2. The number of tubes formed were quantified using an angiogenesis analyzer plugin of ImageJ Software.

### Monocyte migration assay.

Peripheral blood mononuclear cells were isolated from peripheral blood using Ficoll Paque Plus. CD14^+^ cells were isolated using the CD14 MicroBeads. A total of 50,000 CD14^+^ cells were added to the top well of 5 μM porous inserts. Mesothelial cell CM (described above) were added to the bottom wells. Cells were allowed to migrate for 8 hours. The migrated cells were collected and counted using trypan blue (0.4% in PBS) and conventional cell counting methods.

### RNA isolation and quantitative reverse transcription-PCR.

Total RNA was isolated from the primary human mesothelial cells using Trizol (Invitrogen) as per the manufacturer’s protocol. The RNA quality and concentration was determined using the NanoDrop 8000 Spectrophotometer (Thermo Fisher Scientific). Reverse transcription of 2 μg total RNA was carried out using the High-Capacity cDNA Reverse Transcription Kit. Quantitative PCR (qPCR) was performed with predesigned TaqMan probes (Supplemental Table 4) using TaqMan Fast Advanced Master Mix on an Applied Biosystems StepOnePlus Real-Time PCR System (Applied Biosystems). GAPDH was used as a housekeeping gene for normalization. The reactions were run in triplicate with at least 2 biological replicates for each individual experiment. Relative levels of mRNA expression were calculated using the 2^–ΔΔCt^ method. Differences between treatments were evaluated using an unpaired 2-tailed Student’s *t* test.

### RNA-Seq and bioinformatic analysis.

Total RNA was isolated from primary human mesothelial cells 36 hours after treatment with Tyk-nu spheroid CM, ascites, or control media using RNeasy Mini Kit (Qiagen) following manufacturer’s instructions. RNA purity was checked using the NanoPhotometer spectrophotometer (IMPLEN). RNA integrity and quantitation were assessed using the RNA Nano 6000 assay kit of the Bioanalyzer 2100 system (Agilent Technologies). Library preparation and next generation RNA-Seq was carried out by Novogene. Sequencing libraries were generated using NEBNext Ultra RNA Library Prep Kit for Illumina (NEB) and index codes were added to attribute sequences to each sample. The library quality was assessed on the Agilent Bioanalyzer 2100 system. The clustering of the index-coded samples was performed on a cBot Cluster Generation System using PE Cluster Kit cBot-HS (Illumina). After cluster generation, the library preparations were sequenced on an Illumina platform and paired-end reads were generated. Raw reads of FASTQ format were first processed through fastp. In this step, clean reads were obtained by removing reads containing adapter and poly-N sequences and reads with low quality from raw data. At the same time, Q20, Q30, and GC content of the clean data were calculated. All the downstream analyses were based on the clean data with high quality. Paired-end clean reads were aligned to a reference genome using the Spliced Transcripts Alignment to a Reference (STAR) software. FeatureCounts was used to count the read numbers mapped of each gene. The RPKM (reads per kilobase of exon model per million mapped reads) of each gene was calculated based on the length of the gene and reads count mapped to this gene. Differential expression analysis between 2 conditions/groups (3 biological replicates per condition) was performed using DESeq2 R package. The resulting *P* values were adjusted using the Benjamini and Hochberg’s approach for controlling the FDR. Genes with an adjusted *P* value less than 0.05 found by DESeq2 were identified as differentially expressed. Differential expression analysis of 2 conditions without biological replicates was performed using the EdgeR R package. A corrected *P* value of 0.005 and log_2_^(Fold^ ^CHANGE)^ of 1 were set as the threshold for significantly differential expression. Gene set enrichment analysis was performed for 50 Hallmark gene sets using Molecular Signatures Database (MSigDB v7.4). The data were published in the National Center for Biotechnology Information Gene Expression Omnibus database (accession number GSE223165).

### STC1 and ANGTPL4 enzyme-linked immunoassays.

Human primary mesothelial cells were seeded at 4 × 10^5^ cells in 6-well tissue culture plates, cultured for 48 hours, and treated with Tyk-nu spheroid CM, malignant ascites, or control media. For the treatment studies, an IL-6–neutralizing Ab or control IgG (3μg/mL) was added with the malignant ascites. After 36 hours, the media was changed to serum-free media, and the cells were cultured for 24 hours, centrifuged at 500 *g* for 5 minutes, and filtered with a 0.22 μm filter. The concentrations of STC1 and ANGPTL4 were measured using the Human DuoSet ELISA Kits (R&D Systems).

### Ex vivo human adhesion and colonization assays.

A fresh piece of normal human omentum was cut into 8 mm pieces (equivalent weights). For the studies in Figure 2, the tissue was treated with pronase (1 mg/mL in DMEM/F12) for 20 minutes in an orbital shaker at 3.7 *g* at 37°C. The tissue was rinsed with growth media twice and placed in a 24-well dish (1 piece/well). GFP-labeled Kuramochi, OVCAR5 cells, or Tyk-nu (4 × 10^6^ cells/well) were added to each omentum culture and incubated at 37°C in full growth media for 18 hours or 5 days. For the treatment studies, control Abs (IgG1-2 μg/mL and/or IgG2B-200 ng/mL), ANGTPL4 Ab (2 μg/mL), STC1 Ab (200 ng/mL), or both Abs were added at the start of the assay. After incubation, omental tissue explants were washed 3 times in PBS, digested in 5% NP-40 for 30 minutes at 37°C, and scraped with a metal spatula. All cells removed during digestion were placed in a 24-well plate, and the total number of cells was quantified using the PICO imaging cytometer (Molecular Devices).

### Ex vivo mouse adhesion and colonization assays.

Omenta were isolated from C57Bl/6 mice and placed into 96-well dish (1 omentum/well) after treatment with pronase (1 mg/mL in DMEM/F12) for 20 minutes in an orbital shaker at 3.7 *g* at 37°C. GFP-labeled ID8^p53–/–^ cells (1 × 10^6^ cells/well) were added to each omentum culture and incubated at 37°C in full growth media for 18 hours or 5 days. After incubation, omenta were washed 3 times in PBS, digested in 5% NP-40 for 30 minutes at 37°C, and scraped with a metal spatula. All cells removed during digestion were placed in a 24-well plate, and the total number of cells was quantified using the PICO imaging cytometer (Molecular Devices).

### Bioenergetic glycolysis assay.

Glycolysis was measured as previously described (66) with the seahorse Extracellular Flux XF-96 analyzer (Agilent). Briefly, human primary mesothelial cells (with siRNA knockdown of STC1 and ANGPTL-4) were seeded in Seahorse XF-96 plates at a density of 10,000 cells per well and allowed to adhere for 24 hours. The next day media was replaced with either control media or ascites from a patient with high-grade serous OvCa. After 24 hours, cells were changed to unbuffered DMEM without glucose (D5030, Sigma-Aldrich; supplemented with glutamine (2mmol/L), pH adjusted to 7.4 and incubated in a non-CO_2_ incubator for 1 hour. Extracellular acidification rate (ECAR) was determined following sequential injections with D-glucose (10mM), Oligomycin (2μM), and 2-deoxyglucose (100mM). ECAR after injection of D-glucose was a measure of glycolysis and after oligomycin injection represented glycolytic capacity. The glycolytic reserve was quantified by the measure of ECAR after the injection of 2-deoxyglucose. Samples were analyzed with 8 technical replicates and data are representative of 3 independent experiments after normalization using CyQuant cell proliferation assay kit.

### Adhesion assays.

Primary human mesothelial cells were transfected with control, ANGPTL4, or STC1-targeted siRNAs. After 24 hours, 4,000 transfected mesothelial cells were plated on black-walled 384-well plates using the iPipette (Apricot Designs). After another 24 hours, 8,000 fluorescently labeled Tyk-nu-GFP, Ovcar5-GFP, or Kuramochi-GFP OvCa cells were seeded in 40 μL of serum-free media on top of the mesothelial cells (0.33 cm2, *n* = 5–15). After 1-hour incubation at 37°C, the wells were washed with PBS, fixed with 4% paraformaldehyde, and the cell number was computed using the PICO imaging cytometer (Molecular Devices).

### Invasion assays.

Primary human mesothelial cells were transfected with control, ANGPTL4, or STC1-targeted siRNAs. After 24 hours, 10,000 transfected mesothelial cells were plated on precoated (7 μg of collagen type I) 96-well transwell inserts (BD Biosciences; ref. 65). A total of 8,000 Tyk-nu-GFP, Ovcar5-GFP, or Kuramochi-GFP cells were seeded in 40 μL of serum-free media in the upper chamber of a 96-well transwell plate (0.134 cm^2^, *n* = 5–10) precoated with the mesothelial cells (65). The plates were incubated at 37°C for 24–48 hours. All cells were removed from the top chamber, and the invaded OvCa cells were quantified using the PICO imaging cytometer (Molecular Devices).

### Proliferation assay/number of cells over time analysis.

Primary human mesothelial cells were transfected with control, ANGPTL4, or STC1-targeted siRNAs. After 24 hours, 4,000 transfected mesothelial cells were plated on black-walled 384-well plates using the iPipette from Apricot Designs. For Figure 5D and Supplemental Figure 9C, a total of 2,000 Tyk-nu-GFP, 1,000 OVCAR5-GFP, or 4,000 Kuramochi-GFP cells were seeded in 40 μL of growth media on top of the mesothelial cells (*n* = 16). The plates were incubated for 96 hours at 37°C.

### Statistics.

Data were analyzed by GraphPad Prism 8 (Version 9.3.1) and presented as the mean ± SEM of the indicated number of samples. Two-tailed Student’s *t* test, 1-way ANOVA, and 2-way ANOVA were used to determine significance in 2-group and multiple-group experiments. *P* values of less than 0.05 were statistically significant. Pearson’s correlation coefficient analysis was performed on the differential expression values of RNA using Microsoft Excel.

### Study approval.

All animal studies were approved by the IACUC at the University of Chicago. All human tissue collection was approved by the Institutional Review Board, and written informed consent was received prior to collection of tissues.

## Author contributions

HAK, PB, KK, and EL wrote the manuscript. HAK, EL, and PB designed the studies. PB, KK, and HAK conducted the experiments and acquired the data. HAK, PB, KK, AB, KW, and RRL analyzed the data. YR provided mouse models and technical support.

## Supplementary Material

Supplemental data

## Version 1 02/16/2023

In-Press Preview

## Version 2 03/22/2023

Electronic publication
