# Supplementary material for: Cancer-associated mesothelial cell–derived ANGPTL4 and STC1 promote the early steps of ovarian cancer metastasis
Source: JCI Insight. 2023 Mar 22;8(6):e163019. doi: 10.1172/jci.insight.163019 (PMC10070116; doi:10.1172/jci.insight.163019)
Supplement: Supplemental data [file jciinsight-8-163019-s097.pdf]

Supplemental

Title

Cancer-Associated Mesothelial Cell-Derived ANGPTL4 and STC1 Promote the Early Steps of Ovarian Cancer Metastasis

Authors and Affiliations

Preety Bajwa[1], Kasjusz Kordylewicz[1], Agnes Bilecz[1,2], Ricardo R. Lastra[2], Kristen Wroblewski[3], Yuval Rinkevich[4], Ernst Lengyel[1], and Hilary A. Kenny[1]

[1]Department of Obstetrics and Gynecology/Section of Gynecologic Oncology, University of Chicago, Chicago, IL 60637, USA

[2]Department of Pathology, University of Chicago, Chicago, IL 60637, USA

[3]Department of Public Health Sciences, University of Chicago, Chicago, IL 60637, USA

[4]Institute of Regenerative Biology and Medicine, Helmholtz Zentrum München, 81377 Munich, Germany.

Correspondence: Hilary Kenny, Ph.D. hkenny@bsd.uchicago.edu

Supplemental Materials and Methods

Mouse Strains. *Wt1^{GFPCre}* (JAX stock #010911), *and Gt (ROSA)$^{26Sortm1(HBEGF)Awai}$* (JAX stock #007900), commonly known as Rosa26iDTR mice, were obtained from the Jackson Laboratory. *Msln^{CLN}* transgenic mice were a gift from Yuval Rinkevich {Rinkevich, 2012 #4862}. The following primers were used for genotyping: *MslnCLN:* 5' GGA GAA AGC AAG CTC CCA ACT CAT GA 3', 5' CCA CTG CTG TGT TCC AGA AGT GTT GGT 3', and 5' GGG ACA AGT GGG GAC CTC AGA GTC A 3'. *Wt1GFPCre:* 5' CAC TAC CAG CAG AAC ACC CCC ATC 3', 5' TTG CGA ACC TCA TCA CTC GTT GC 3', 5' GGC TTA AAG GCT AAC CTG GTG TG 3', 5' GGA GCG GGA GAA ATG GAT ATG 3'.

To establish the MSLN-iDTR mouse model, *Msln*^{CLN} mice were crossed with Rosa26iDTR mice. Littermates were assigned randomly to the experimental groups. Animals were housed under specific pathogen-free conditions with *ad libitum* access to food and water. All procedures involving animal care were approved by the Institutional Care and Use Committee at the University of Chicago (Chicago, IL).

Primary human tissue collection and primary cell culture. Specimens of fresh human omentum were obtained from consented patients undergoing surgery for benign conditions. Specimens of fresh human omental metastases and malignant ascites were obtained from consented patients undergoing surgery for OvCa. The protocols were approved by the University of Chicago Institutional Review Board. Primary human mesothelial cells were isolated from normal human omentum as previously described and were used within two passages of isolation {Kenny, 2007 #1932;Peters, 2015 #5064}. Primary human monocytes were isolated from human peripheral blood using positive immunomagnetic separation with CD14 microbeads (Miltenyi Biotec) following the manufacturers' protocol.

siRNA transfections. Human primary mesothelial cells were transfected with STC1 siRNA (25 nm), ANGPTL-4 siRNA (25 nm), or negative control siRNA (20 nm) using Lipofectamine 2000 for 24 hours, followed by treatment with control media, Tyk-nu conditioned media, or human ascites. For the IL-6 blocking studies, an IL-6 neutralizing antibody or control IgG (3μg/ml) was added with the malignant ascites. Forty-eight hours after treatment, the cells were collected for mRNA and protein isolation.

Generation of conditioned medium and cell treatments. For the generation of OvCa spheroid conditioned media, 3×10^6 Tyk-nu cells were grown in ultra-low attachment plates (75cm^2) for 72 hours at 5% CO_2, 37ºC. The cell supernatants were collected after centrifugation, filtered through a 0.45 μm filter, and immediately used or stored at -80°C for later use. For the generation of primary human mesothelial cell-conditioned media, cells were seeded at 4×10^5 cells in 6-well tissue culture plates and incubated at 37°C until cuboidal morphology and cell confluence was reached. The mesothelial cells were then treated with control media, Tyk-nu conditioned media, or human ascites. Thirty-six hours later, the media was changed to serum-free media, and the cells were incubated at 5% CO_2, 37ºC. Twenty-four hours later, the media was collected, centrifuged 0.5g x 5 minutes, filtered through 0.22 μm filter, and immediately used for endothelial and cancer cell functional assays.

Ex-vivo human colonization assays. A fresh piece of normal human omentum was cut into 8 mm pieces (equivalent weights). For the treatment studies, control antibodies (IgG1-2 μg/ml and/or IgG2B-200 ng/ml), ANGTPL4 antibody (2 μg/ml), STC1 antibody (200 ng/ml) or both antibodies were added at the start of the assay. After incubation for 5 days, omental pieces were washed 3 times in PBS, digested in 5% NP-40 for 30 minutes at 37°C, and scraped with a metal spatula. After digestion, the unlabeled cells were collected by fluorescently-activated cell sorting using the Bigfoot (Invitrogen) and qRT-PCR was performed

In vivo adhesion and colonization assays. For the studies in Supplemental Figure 2-3, heterozygous MSLN-iDTR mice (expressing the Msln-, Cre- LacZ-, and diphtheria toxin receptor) or wild-type littermates were randomized into experimental groups (*n* = 8-10 mice/group). A tamoxifen diet (400mg/kg; Envigo) was provided *ad libitum* for 14 days. On day 15, all mice received a regular chow diet, and the mice received intraperitoneal injections of diphtheria toxin (100 ng/100μl) or solvent control (PBS) every 24 hours for 3 days. On the 3rd day, the mice were injected intraperitoneally with 5 × 10^6 ID8$^{p53-/-Brca2-/-}$ pchili/luciferase cells in cold-PBS (500 μL total).

Mouse tissue immunohistochemistry and immunofluorescence. Harvested mouse omental tumors were formalin fixed prior to paraffin embedding. The fixed tissues were dehydrated using increasing dilutions of ethanol, cleared in xylene, embedded in paraffin wax, and 5 μm thick sections were mounted on Superfrost Plus charged slides (Thermo Fisher Scientific). The slides were deparaffinized in xylene and rehydrated in decreasing dilutions of ethanol. Antigen retrieval was performed in 10 mmol/L sodium citrate buffer (0.05% Tween-20, pH 6) for 30 minutes at 100°C followed by incubation with endogenous peroxide block using 3% (v/v) hydrogen peroxide in absolute methanol for 20 minutes at room temperature. The slides were blocked in 10% normal goat serum in PBS, 0.1% Triton-X 100, and incubated overnight at 4°C with primary antibodies against GFP (1:100). The staining was visualized using a ready to use VECTASTAIN Elite ABC-HRP kit and a DAB Substrate kit and counterstained with hematoxylin. Images were photographed using a Nikon Eclipse Ti2 (Nikon) microscope.

For immunofluorescence, all slides were blocked in 10% normal goat serum in PBS, 0.1% Triton-X 100 after the antigen retrieval step and incubated overnight at 4°C with primary antibodies against PDPN (1:100) and CK-19 (1:100) followed by incubation with Alexafluor 488 or Alexafluor 568- labeled secondary antibodies (1:250). Nuclei were stained with Hoechst 33342 nucleic acid stain (1:1000 in 1% BSA in PBST) and slides were washed and mounted with ProLong Gold Antifade. Appropriate negative controls for the immunostaining were prepared by omitting the primary antibody. Images were photographed using a live cell DSU Spinning Disk Confocal microscope (Olympus).

RNA isolation and Quantitative Reverse Transcription- Polymerase Chain Reaction. Total RNA was isolated from the primary human mesothelial cells using Trizol (Invitrogen) as per the manufacturer's protocol. The RNA quality and concentration was determined using the NanoDrop 8000 Spectrophotometer (Thermo Fisher). Reverse transcription of 2 μg total RNA was carried out using the High-Capacity cDNA Reverse Transcription Kit. qPCR was performed with predesigned TaqMan probes (Supplemental Table 4) using TaqMan Fast Advanced Master Mix on an Applied Biosystems StepOnePlus Real-Time PCR System (Applied Biosystems). GAPDH was used as a housekeeping gene for normalization. The reactions were run in triplicate with at least 2 biological replicates for each individual experiment. Relative levels of mRNA expression were calculated using the $2^{-\Delta\Delta Ct}$ method. Differences between treatments were evaluated using an unpaired two-tailed Student's *t*-test.

RNA-seq and bioinformatic analysis. Total RNA was isolated from primary human mesothelial cells 36 hours after treatment with Tyk-nu spheroid conditioned media, ascites, or control media using the RNeasy Mini kit (Qiagen) following manufacturer's instructions. RNA purity was checked using the NanoPhotometer® spectrophotometer (IMPLEN). RNA integrity and quantitation were assessed using the RNA Nano 6000 assay kit of the Bianalyzer 2100 system (Agilent Technologies, CA, USA). Library preparation and next generation RNA sequencing was carried out by Novogene. Sequencing libraries were generated using the NEBNext® Ultra™ RNA library Prep kit for Illumina® (NEB, USA) following manufacturer's recommendations and index codes were added to attribute sequences to each sample. The library quality was assessed on the Agilent Bioanalyzer 2100 system. The clustering of the index-coded samples was performed on a cBot Cluster Generation System using the PE Cluster Kit cBot-HS (Illumina) according to the manufacturer's instructions. After cluster generation, the library

preparations were sequenced on an Illumina platform and paired-end reads were generated. Raw data (raw reads) of the FASTQ format were first processed through fastp. In this step, clean reads were obtained by removing reads containing adapter and poly-N sequences and reads with low quality from raw data. At the same time, Q20, Q30 and GC content of the clean data were calculated. All the downstream analyses were based on the clean data with high quality. Paired-end clean reads were aligned to the reference genome using the Spliced Transcripts Alignment to a Reference (STAR) software. FeatureCounts was used to count the read numbers mapped of each gene. RPKM (Reads Per Kilobase of exon model per Million mapped reads) of each gene was calculated based on the length of the gene and reads count mapped to this gene. Differential expression analysis between two conditions/groups (three biological replicates per condition) was performed using DESeq2 R package. The resulting *P* values were adjusted using the Benjamini and Hochberg's approach for controlling the False Discovery Rate (FDR). Genes with an adjusted *P* value < 0.05 found by DESeq2 were identified as differentially expressed. Differential expression analysis of two conditions without biological replicates was performed using the EdgeR R package, A corrected p-value of 0.005 and $\log_2{}^{(\text{Fold CHANGE})}$ of 1 were set as the threshold for significantly differential expression. Gene set enrichment analysis was performed for 50 Hallmark gene sets using the Molecular Signatures Database (MSigDB v7.4).

Hypoxia. Ten thousand primary human mesothelial cells were plated in black-walled 96-well plates and incubated at 37°C for 24 hours. The mesothelial cells were transfected with control, ANGPTL4, or STC1-targeted siRNAs (as described above). The mesothelial cells were then treated with malignant ascites or control growth media and cultured at 5%CO_2, 37ºC. Forty-eight hours later, the medium was replaced with fresh growth medium containing Image-iT Green Hypoxia Reagent at a final concentration of 5 µM, and the cells were incubated at 5%CO_2/37ºC. The cells were washed with PBS and fixed in 4% paraformaldehyde containing Hoechst 33342 (2 µM) for 10 minutes. The paraformaldehyde was exchanged with PBS. The fluorescence was quantified using the SpectraMax iD5 Microplate Reader (Molecular Devices).

Bioenergetic Glycolysis assay. Glycolysis was measured as previously described (60) with the seahorse Extracellular Flux XF-96 analyzer (Agilent). Briefly, human primary mesothelial cells were seeded in Seahorse XF-96 plates at a density of 10,000 cells per well and allowed to adhere for 24 hours. The next day, media was replaced with either control media or ascites from a patient with high grade serous OvCa, and control antibodies, ANGTPL4 antibody (2 µg/ml), STC1 antibody (200 ng/ml) or both antibodies were added to the assay. After 24 hours, cells were changed to unbuffered DMEM without glucose (D5030, Sigma-Aldrich supplemented with glutamine (2mmol/L), pH adjusted to 7.4, and incubated in a non-CO_2 incubator for 1 hour. The extracellular acidification rates (ECAR) were determined following sequential injections with D-glucose (10mM), Oligomycin (2µM), and 2-deoxyglucose (100mM). The extracellular acidification rates (ECAR) after the injection of D-glucose were a measure of glycolysis and after the injection of oligomycin represented glycolytic capacity. The glycolytic reserve is quantified by the measure of ECAR after the injection of 2-deoxyglucose. Samples were analyzed with 8 technical replicates. Data is representative of 3 independent experiments after normalization using the CyQuant cell proliferation assay kit.

Statistics. Data were analyzed by GraphPad Prism 8 (Version 9.3.1) and presented as mean± standard error mean (SEM) of the indicated number of samples. Student's t -test two-tailed and one-way ANOVA with pairwise comparisons were used to determine significance in two-group and multiple-group experiments, respectively. *P* values of less than 0.05 were statistically significant.

Supplemental Figures

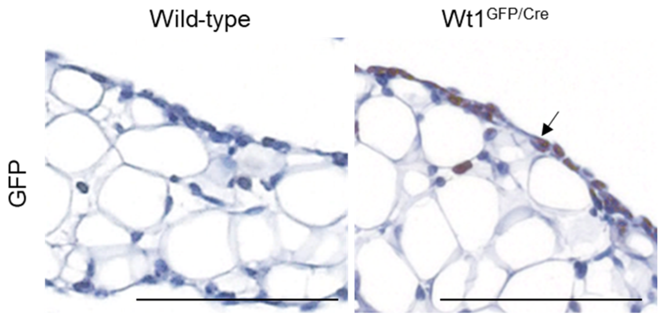

Supplemental Figure 1. Tracing mesothelial cells. GFP expression in mesothelial cells of mouse omentum from a transgenic mouse. Immunohistochemical localization of GFP-labeled mesothelial cells in mouse omentum collected from C57BL/6 (wild-type) or heterozygous *Wt1$^{tm1(EGFP/Cre)Wtp}$/J* mice. Black arrow, GFP-expressing mesothelial cell. Scale bar 500 µm.

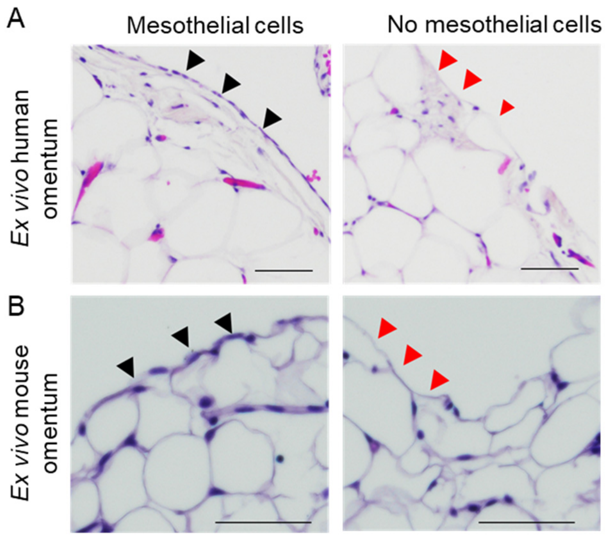

Supplemental Figure 2. Depletion of mesothelial cells from human and mouse omentum *ex vivo*. A.-B Hematoxylin and eosin images of human (**A**) or C57Bl/6 mouse (**B**) *ex-vivo* omentum digested with pronase. Black arrowheads, mesothelial cells. Red arrowheads, site of digested mesothelial cells. Scale bar 500 μm.

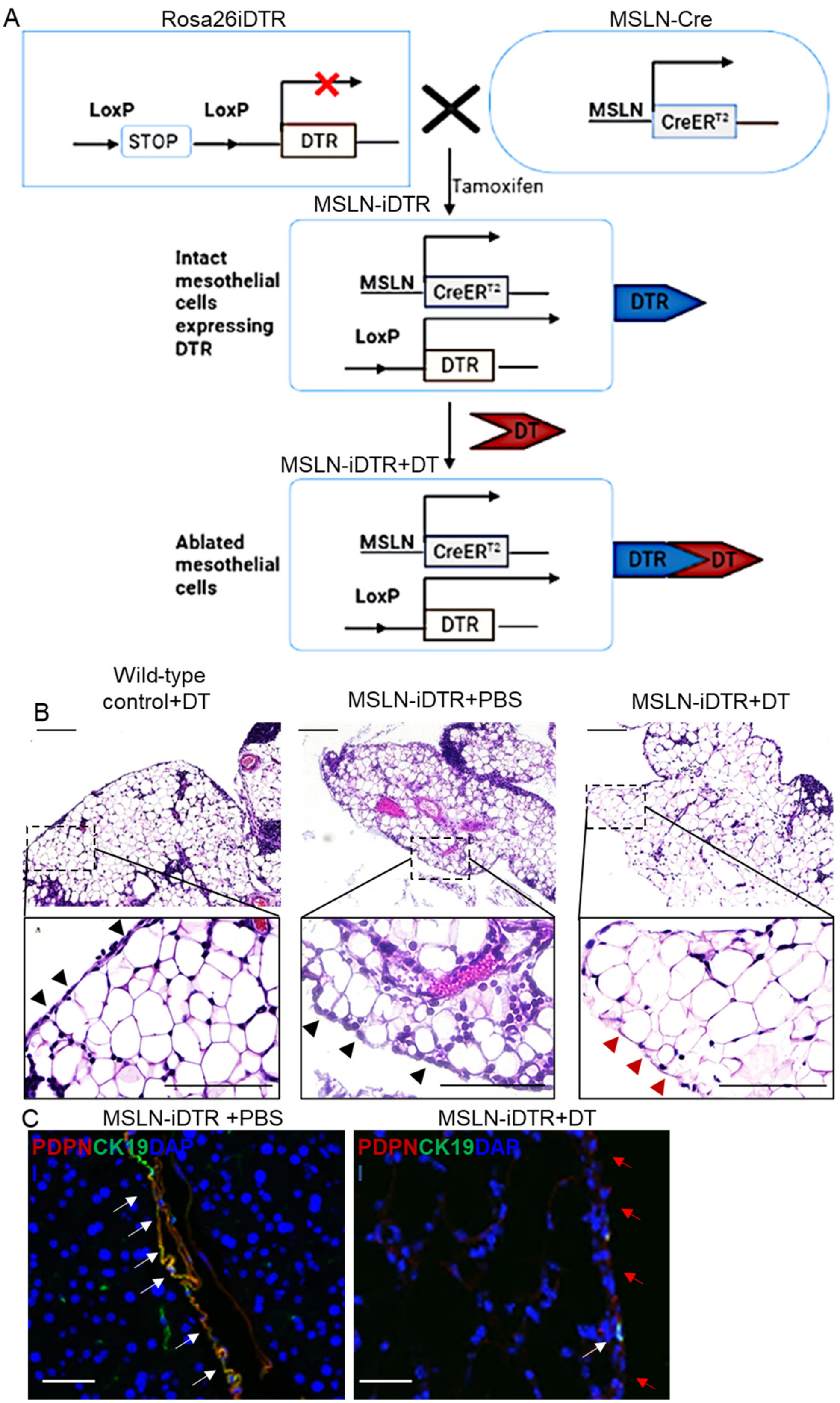

Supplemental Figure 3. Establishment of a mouse model for inducible mesothelial cell depletion. A. Schematic illustrating the ablation of mesothelial cells using the inducible diphtheria toxin receptor (iDTR) system. Crossing the MSLN-Cre^{ERT2} strain to Rosa26iDTR strain results in mice that express DTR under the control of the mesothelin promoter (MSLN-DTR) when fed a tamoxifen diet. Mesothelial cell ablation is obtained

after three intra-peritoneal injections of diptheria toxin (DT) in heterozygous mice. **B**. Representative images of hematoxylin and eosin-stained omental tissues from wild-type littermate mice (control) post DT injection, MSLN-DTR mice post PBS injection, or MSLN-DTR mice post DT injection. Black arrowhead, mesothelial cells. Red arrowheads denuded mesothelial cells. PBS, phosphate-buffered saline. Size bar, 500 μm. **C**. Representative images of immunofluorescence for PDPN (red) and CK19 (green) in MSLN-DTR mice omentum post DT or PBS injection. The counterstain, Hoechst (blue), used to label nuclei. White arrows, mesothelial cells. Red arrows, site of ablated mesothelial cells. Scale bar, 200 μm.

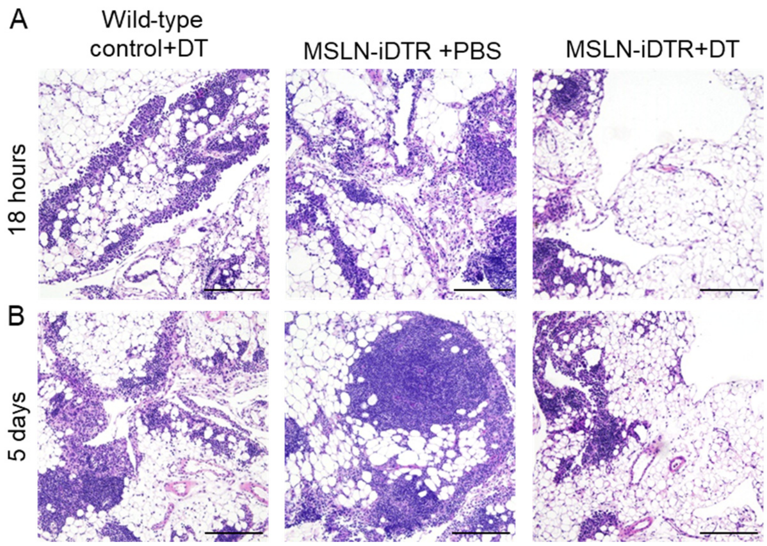

Supplemental Figure 4. Ovarian cancer cell metastasis in an inducible mesothelial cell depletion mouse model. A-B. Hematoxylin and eosin-stained mouse omentum from diptheria toxin (DT) -treated C57BL/6 (wild-type control), DT-treated MSLN-iDTR or phosphate-buffered saline (PBS) -treated MSLN-iDTR mice 18 hours (**A**) or 5 days (**B**) post intraperitoneal-injection of ID8$^{p53-/-Brca2-/-}$ mouse ovarian cancer cells (5 x 10^6). Size bar, 500 µm.

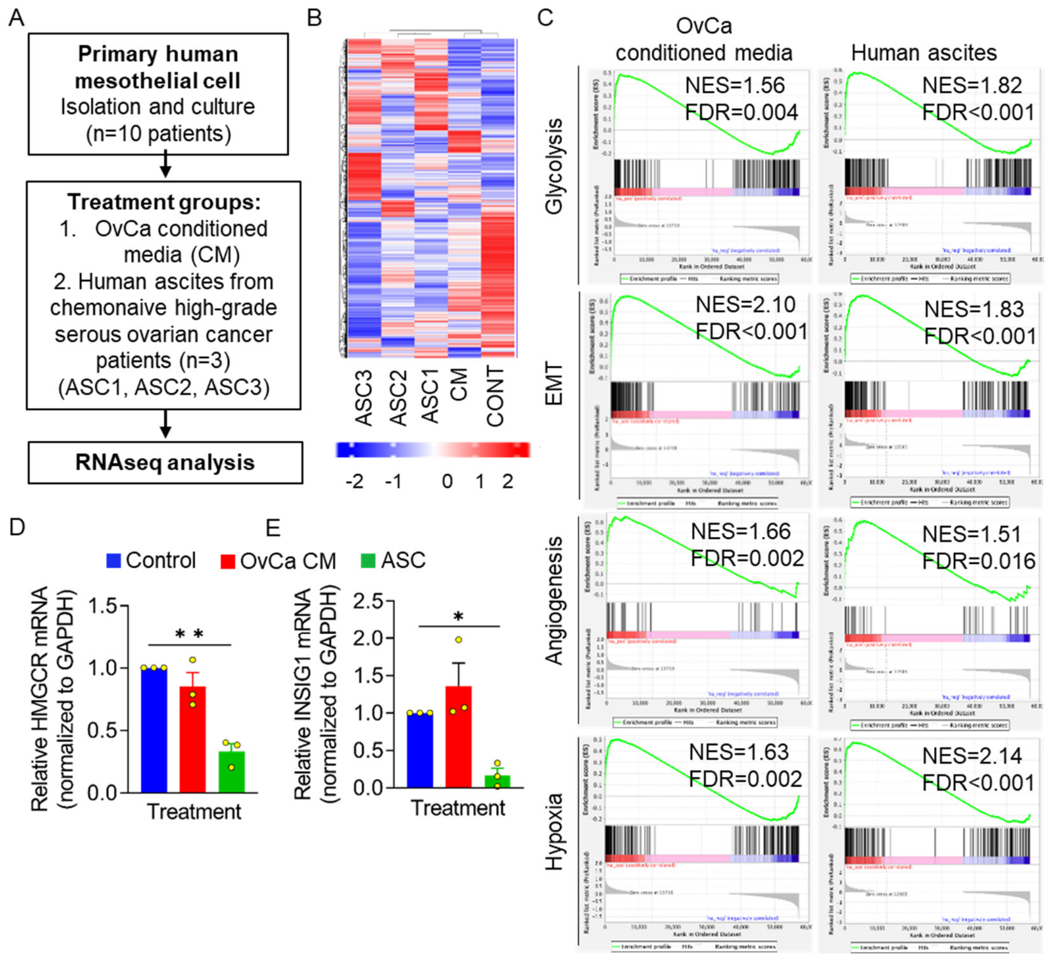

Supplemental Figure 5. Gene set enrichment analysis of transcriptomic changes in by ovarian cancer cells in primary human mesothelial cells. A. Workflow of the transcriptomic analysis performed on human primary mesothelial cells after 36 hours treatment with control media (Control), Tyk-Nu spheroid conditioned media (CM), or ASC (1, 2 or 3). **B**. Differentially expressed genes (DEGs) between different treatment groups shown in a hierarchical clustering heatmap. Rows are expression levels denoted as the z-score, displayed in a high-low (red-blue) color scale. The numeric scale indicates z-transformation. **C**. Gene set enrichment analysis plots demonstrating normalized enrichment score (NES) of the RNA-seq data. The common pathways that both CM (top) and ASC (bottom) regulate are shown. FDR, false discovery rate. **D-E**. Expression levels of HMGCR (**D**) or INSIG1 (**E**) mRNA in mesothelial cells treated with CM or ASC were measured using qRT-PCR. Data is represented mean±SEM (n=3). *, $p<0.05$ and **, $p<0.01$ calculated by one-way ANOVA.

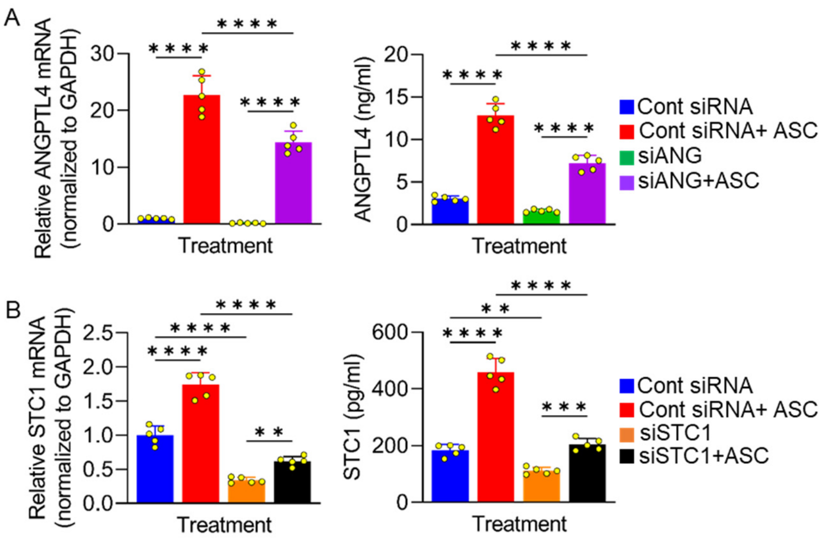

Supplemental Figure 6. Confirmation of transient knockdown of ANGPTL4 AND STC1 in primary human mesothelial cells. A-B. The expression and secretion levels of ANGPTL-4 (**A**) and STC1 (**B**) from mesothelial cells was measured using qRT-PCR (left) or enzyme linked immunoassays (ELISA, right). Mesothelial cells were transfected with control, STC-1, or ANGPTL-4 specific siRNA and treated with human ascites (ASC). After 48 hours qRT-PCR was performed, or mesothelial cell conditioned media was collected for ELISA analysis. Data shown as mean±SEM (n=5). *, $p<0.05$ and **, $p<0.01$ calculated by one-way ANOVA

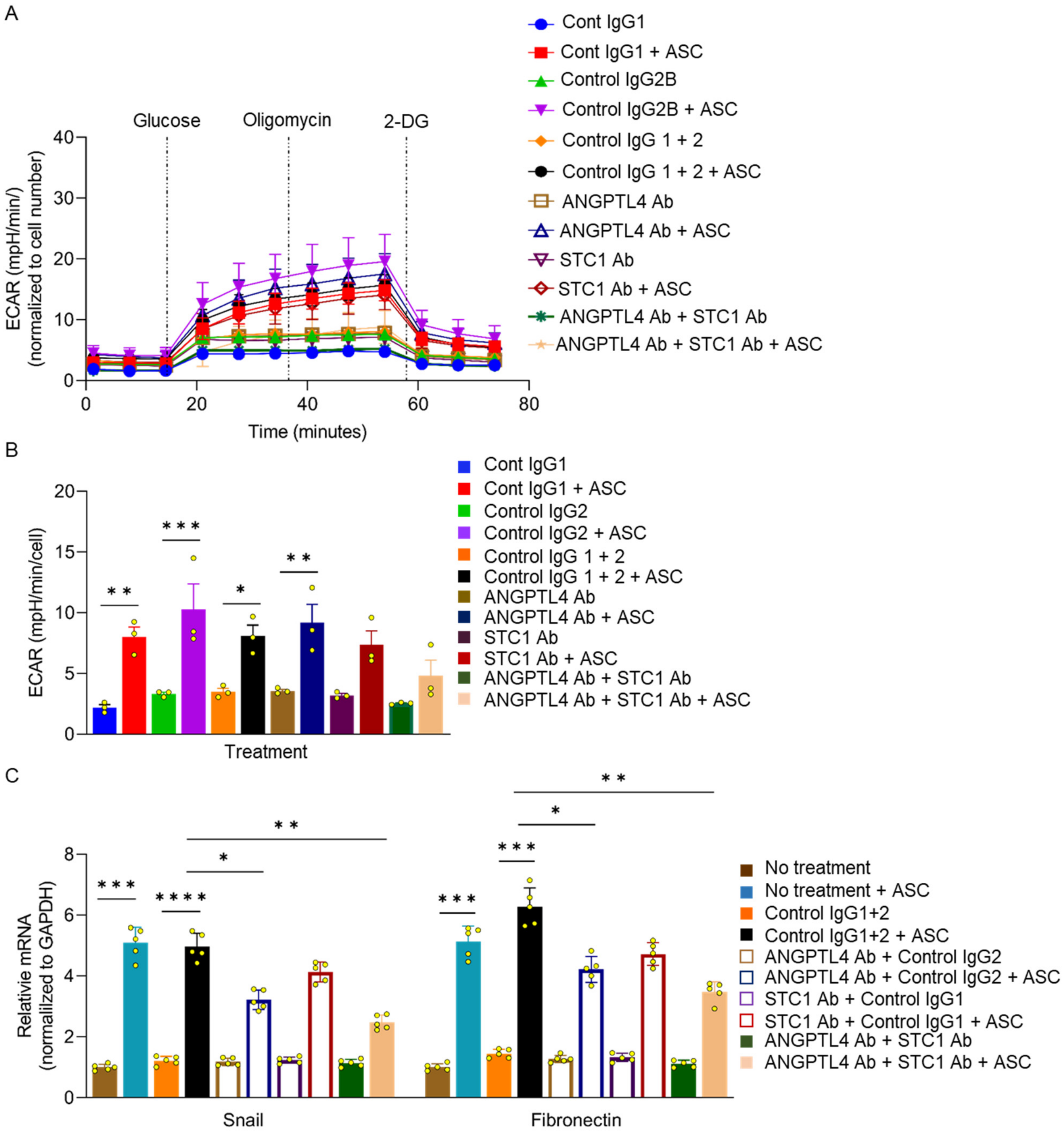

Supplemental Figure 7. Combination treatment with ANGPTL4 and STC1 neutralizing antibodies has effect on ascites-induced glycolysis in mesothelial cells. A-B. Seahorse glucose metabolism in mesothelial cells was measured. Primary human mesothelial cells were treated with control (IgG1 and/or IgG2) STC1 or ANGPTL4 specific neutralizing antibodies and stimulated with human ascites (ASC) or control media (48 hours). **A**. The extracellular acidification rate (ECAR) profile of mesothelial cells following glucose, oligomycin, and 2-DG treatments. **B**. Changes in basal ECAR, glycolytic capacity and glycolytic reserve in the mesothelial cells. Data represented as Mean±SEM (n=3). *, p<0.05, **, p<0.01, and ***, p<0.001 comparison by one-way ANOVA. **C**. Mesothelial cells were treated with control (IgG1 and/or IgG2) STC1 or ANGPTL4 specific neutralizing antibodies and stimulated with human ascites (ASC) or control media (36 hours). qRT-PCR for mesenchymal (snail and fibronectin) markers. Data shown as mean±SEM (n=5). *, p<0.05, **, p<0.01, ***, p<0.001, and ****, p<0.0001 calculated by one-way ANOVA.

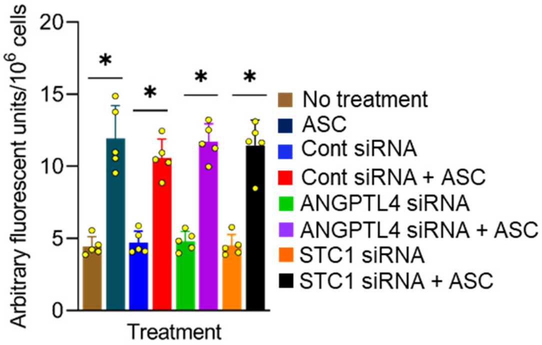

Supplemental Figure 8. Ascites-induced hypoxia is not regulated by ANGPTL4 or STC1. Hypoxia was measured in mesothelial cells using the Image-iT Green Hypoxia Reagent. The nuclei were stained with Hoechst. Primary human mesothelial cells were transfected with control, ANGPTL4 or STC1 specific siRNA and 24 hours later treated with human ascites (ASC) or control media for 48 hours. Data represented as mean±SEM (n=5). *, p<0.05 calculated by one-way ANOVA.

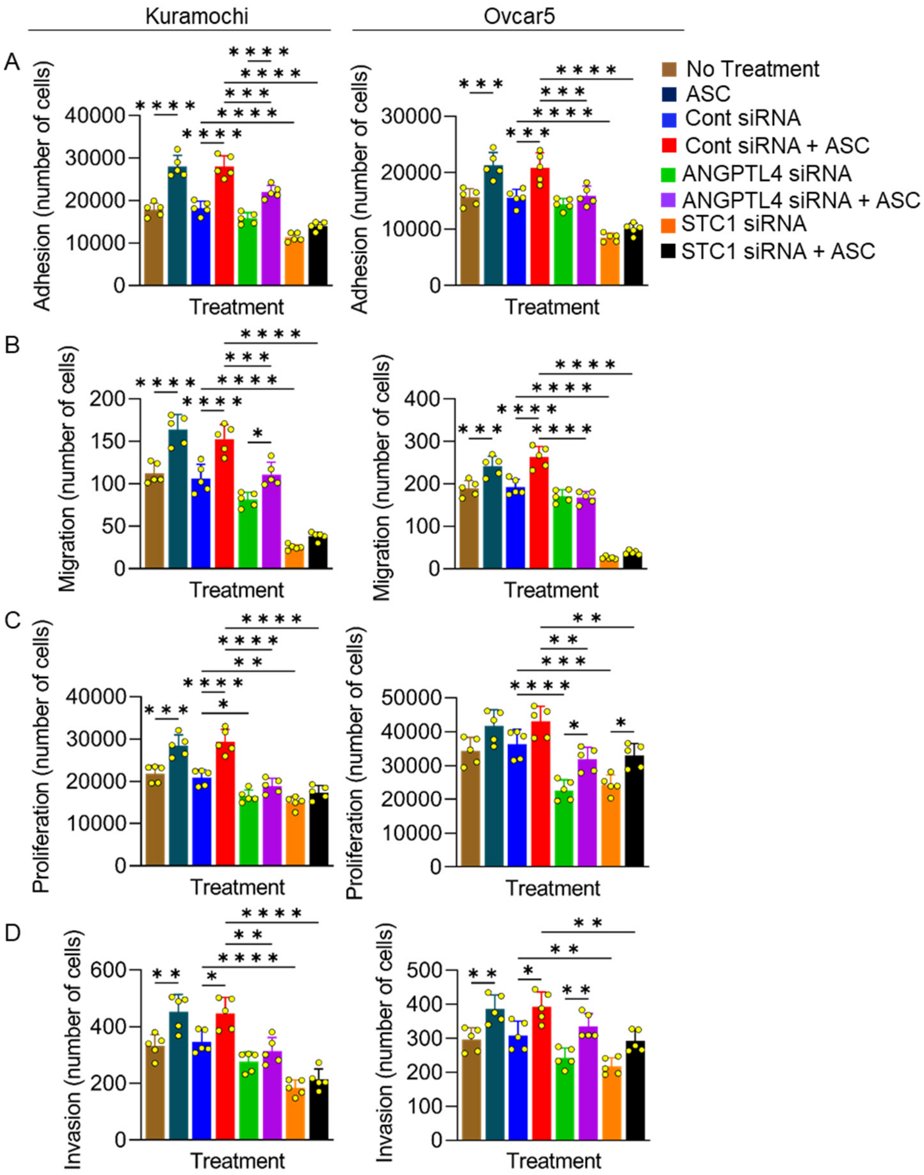

Supplemental Figure 9. Mesothelial cell derived ANGTPLT4 and STC1 regulate ovarian cancer cell function. Mesothelial cells were treated with control siRNA or siRNA targeting STC-1 or ANGPTL4 followed by treatment with ascites (ASC) for twenty-four hours. Functional assays were performed using fluorescently labeled Kuramochi or Ovcar5 ovarian cancer (OvCa) cells. **A**. OvCa cell adhesion (1 hour) was tested on top of the transfected and pre-treated mesothelial cell monolayer. **B**. OvCa cell migration (12-24 hours) was tested using a Boyden chamber lined with transfected mesothelial cells. **C**. OvCa cell proliferation (72 hours) was tested on top of the transfected and pre-treated mesothelial cell monolayer. **D**. OvCa cell invasion (24-48 hours) was tested through transfected and pre-treated mesothelial cell monolayer and rat tail collagen type I using a Boyden chamber. All data shown as Mean ± SEM (n=5). *, $p<0.05$, **, $p<0.01$, ***, $p<0.001$, and ****, $p<0.0001$ calculated by one-way ANOVA.

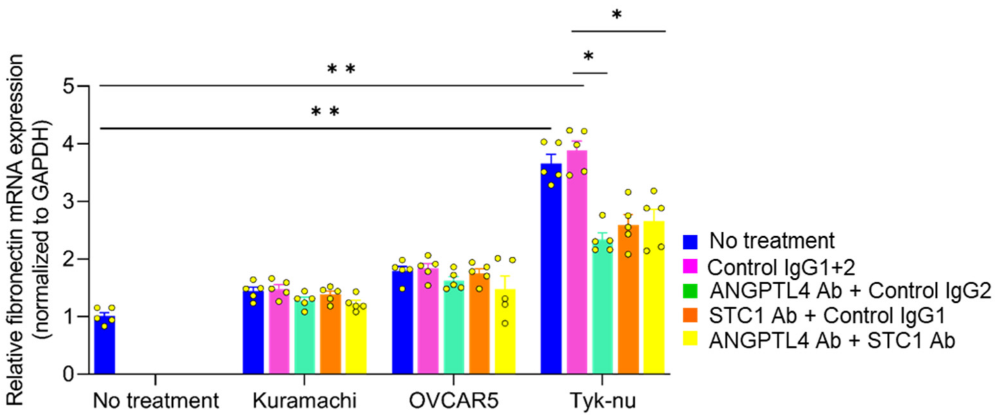

Supplemental Figure 10. Treatment with an ANGPTL4 neutralizing antibody inhibits Tyk-nu cell induced fibronectin expression on the surface cells of the human omentum. Human *ex-vivo* colonization assay. GFP-labeled Tyk-nu, OVCAR5 or Kuramochi, OvCa cells were seeded on human omental tissue explants. The cultures were untreated or treated with an ANGPTL4 neutralizing antibody, a STC1 neutralizing antibody, the respective control IgGs, or both neutralizing antibodies. The cancer cells and mesothelial cells were digested off the omentum and the unlabeled omental surface cells were collected using fluorescently-activated cell sorting and qRT-PCR was performed. Data represented as mean ±SEM (n=8 patients/ group). *, p<0.05 and **, p<0.01 by one-way ANOVA.

Supplemental Tables

Supplemental Table 1. Genes regulated in mesothelial cells by ascites and OvCa-conditioned media (Top 100 genes compared, all positively regulated)

Gene	Gene Name	Fold Change (log2)	P-value (-log10)	Fold Change (log2)	P-value (-log10)	Fold Change (log2)	P-value (-log10)	Fold Change (log2)	P-value (-log10)
		**ASC1**		**ASC2**		**ASC3**		**CM**	
STC1	Stanniocalcin 1	3.40	14.18	2.72	13.06	3.59	28.64	2.55	22.02
PI3	Peptidase inhibitor 3	4.39		3.03	14.03	3.74	19.34	2.91	10.30
PGK1	Phosphoglycerate kinase 1	1.49	10.54	1.19	8.57	1.43	19.23	0.69	8.38

Supplemental Table 2. Genes regulated in mesothelial cells by ascites (Top 100 genes compared, increased and decreased)

Gene	Gene Name	Fold Change (log2)	P-value (-log10)	Fold Change (log2)	P-value (-log10)	Fold Change (log2)	P-value (-log10)
		ASC1		ASC2		ASC3	
ANGPTL4	Angiopoietin-like 4	6.689	38.41	4.88	34.17	5.90	49.48
STC1	Stanniocalcin 1	3.40	14.18	2.72	13.06	3.59	28.64
PI3	Peptidase inhibitor 3	4.39	11.57	3.03	14.04	3.74	19.34
SBNO2	Strawberry notch homolog 2	0.97	13.34	0.73	8.48	1.00	22.65
PGK1	Phosphoglycerate kinase 1	1.49	10.54	1.19	8.57	1.43	19.23
NAMPT	Nicotinamide phosphoribosyltransferase	2.05	10.81	1.86	10.79	1.84	14.61
GPI	Glucose-6-phosphate isomerase	1.21	10.17	1.03	15.64	1.04	15.08
INSIG1	Induced insulin gene 1	-2.95	17.92	-2.30	25.43	-2.82	31.40
MVK	Mevalonate kinase	-1.61	25.15	-1.59	25.15	-1.86	30.83
HMGCS1	3-Hydroxy-3-methylglutaryl-coA synthase 1	-2.72	27.04	-2.55	29.07	-2.63	25.35
IDI1	Isopentenyl-diphosphate delta isomerase 1	-1.96	21.93	-1.86	19.01	-1.94	23.96
FADS1	Fatty acid desaturase 1	-1.30	13.73	-1.42	19.24	-1.42	23.84
IDH1	Isocitrate dehydrogenase 1	-1.02	10.85	-0.90	12.46	-1.23	23.60
LPIN1	Lipin 1	-1.17	27.89	-1.13	18.53	-1.26	23.07
HMGCR	3-Hydroxy-3-methylglutaryl-coA reductase 1	-1.48	19.17	-1.63	19.43	-1.59	20.56
MMAB	Metabolism of cobalamin associated B	-1.50	14.68	-1.46	11.69	-1.96	20.41
MSMO1	Methylsterol monooxygenase 1	-1.89	14.43	-2.00	20.40	-2.01	15.38
PCSK9	Proprotein convertase subtilisin/kexin type 9	-3.31	10.62	-4.27	13.42	-4.58	15.17
MVD	Diphosphomevalonate decarboxylase	-1.31	11.01	-1.25	12.78	-1.38	14.70
FDFT1	Farnesyl-diphosphate farnesyltransferase 1	-1.31	16.27	-1.24	15.70	-1.23	14.27
LDLR	Low density lipoprotein receptor	-1.40	11.95	-1.68	18.55	-1.61	13.91

Supplemental Table 3. Description of reagents and resources.

REAGENT OR RESOURCE	SOURCE	IDENTIFIER
**Antibodies**		
Anti- β-Actin	Sigma-Aldrich	A5441
Anti- Mesothelial cell (HBME-1)	Agilent	M3505
Anti-GFP (D5.1)	Cell Signaling	2956
Anti-PDPN	Thermo Fisher	14538182
Anti-mcherry	Abcam	Ab167453
Anti-Cytokeratin 19	Abcam	Ab52625
Anti-STC1	Novus Biologicals	NBP2-97047
Anti-ANGPTL4	Proteintech	18374-1-AP
Anti-Calretinin	Thermo Fisher	180211
Anti-Snail (C15D3)	Cell Signaling	3879T
Anti-E-cadherin	Cell Signaling	3195
anti-rabbit IgG-HRP	Cell Signaling	7074
anti-mouse IgG-HRP	Cell Signaling	7076
Alexa Fluor 488 goat anti-rabbit IgG (H+L)	Thermo Fisher	A32731
Alexa Fluor 594 goat anti-hamster IgG (H+L)	Thermo Fisher	A11012
Alexa Fluor 568 goat anti-hamster IgG (H+L)	Thermo Fisher	A21112
ANGPTL4 blocking (monoclonal antibodies against mouse and human)	Gift from Professor Andrew Tan Nguan Soon	Nanyang Technological University, Singapore
Anti-STC1	R & D Systems	MAB2958
IL-6 blocking	R & D Systems	MAB206
**Chemicals and Recombinant Proteins**		
FB-Essence	VWR	10803-034
FBS	Corning	35010CV
RPMI1640	Corning	10-040-CV
DMEM- without glucose	Sigma-Aldrich	D5030
DMEM	Corning	10-013-CV
Pronase	Sigma-Aldrich	10165921001
Hydrocortisone solution	Sigma-Aldrich	H6909-10ML
Insulin-Transferrin-Selenium-X	Life Technologies	41400-045
Hoechst 33258 Pentahydrate (bis-Benzamide)	Thermo Fisher	H3569
Penicillin/Streptomycin	Corning	30-002-CI
Antibiotic-Antimycotic	Life Technologies	15240-062
MEM non-essential amino acids	Corning	25-025-CI
MEM vitamins	Corning	25-020-CI
IL-6	Peprotech	200-06
Glucose	Sigma-Aldrich	G7021
Oligomycin A	Sigma-Aldrich	75351
2-Deoxy-D-glucose	Sigma-Aldrich	D8375
Trypan Blue Solution, 0.4%	Thermo Fisher/Gibco	15250061
CFSE	Thermo Fisher	65-0850-84
Lipofectamine-2000	Thermo Fisher	11668500
Tamoxifen	Envigo	
Prolong Gold Antifade Mountant	Thermo Fisher	P36930
Promega Luciferase Assay System	Thermo Fisher	E4030
VECTASTAIN Elite ABC-HRP Reagent	Vector Laboratories	PK-7100
DAB Substrate Kit, Peroxidase (HRP), with Nickel, (3,3'-diaminobenzidine)	Vector Laboratories	SK-4100
**Critical Commercial Assays**		
Human Cytokine Array Kit	R & D Systems	ARY005B
ANGPTL4 ELISA	R & D Systems	DY3485
STC1 ELISA	R & D Systems	DY2958
RNeasy Mini Kit	Qiagen	74104
Image-iT Green Hypoxia Reagent	Thermo Fisher	I14833
CyQuant cell proliferation assay kit	Thermo Fisher	C7026

Dual-Luciferase Reporter Assay Systems	Promega	E1910
Angiogenesis Starter Kit	Thermo Fisher	A1460901
CD14 microbeads	Militenyi Biotec	130-050-201
Ficoll® Paque	Cytiva	17-1440-02
**siRNAs**		
ON-Targetplus siRNA- SMARTpool human STC1	Dharmacon	L-006477-00-0005
ON-Targetplus siRNA- SMARTpool human ANGPTL4	Dharmacon	L-007807-00-0050
siGENOME non-targeting control siRNA pool #1	Dharmacon	D-001206-13-05
**Recombinant DNA**		
pLenti6-H2B-mcherry	Addgene	Plasmid #89766
pUltra-Chili-Luc	Addgene	Plasmid #48688
**Biological Samples**		
Primary human omental tissue	University of Chicago, Chicago, IL, USA	IRB #13372B
Human ascites	University of Chicago, Chicago, IL, USA	IRB #13372B
Mouse omentum	University of Chicago, Chicago, IL, USA	IACUC #71921
**Experimental Models: Cell Lines**		
TYK-nu	University of California Los Angeles, CA, USA	Koneczny Laboratory
OVCAR5	UCSF,San Francisco, USA	
Kuramochi	JCRB cell bank, Japan	#JCRB0098
ID8p53$^{-/-}$Brca2$^{-/-}$	Imperial College London, London, UK	McNeish Laboratory
ID8p53$^{-/-}$Brca2$^{-/-}$ cGFP	University of Chicago, Chicago, IL, USA	
ID8p53$^{-/-}$Brca2$^{-/-}$ mcherry	University of Chicago, Chicago, IL, USA	
ID8p53$^{-/-}$Brca2$^{-/-}$ pchili/luciferase	University of Chicago, Chicago, IL, USA	
**Experimental Models: Organisms and Strains**		
$Wt1^{tm1(EGFP/cre)Wtp}$/J	The Jackson Laboratory	#010911
$Msln^{CLN}$	Helmholtz, Munich, Germany	Rinkevich laboratory
C57BL/6-$Gt\ (ROSA)26Sor^{tm1(HBEGF)Awai}$/J (ROSA26iDTR)	The Jackson Laboratory	#007900
C57BL/6NCrl	Charles River	027
**Software and Algorithms**		
Prism Software	GraphPad	RRID: SCR_002798
ImageJ	https://imagej.nih.gov/ij/	RRID: SCR_003070
Fiji	https://imagej.net/software/fiji/	RRID:SCR_002285
Molecular Signatures Database	Broad Institute, MIT	RRID: SCR_016863
Qupath	https://qupath.github.io/	
Biorender	https://biorender.com/	Used for creating graphical abstract

Supplemental Table 4. List of TaqMan Probes.

Gene of Interest	Abbreviation	Assay ID
Macrophage migration inhibitory factor	MIF	Hs00236988_g1
Chemokine (C-X-C motif) ligand 1	CXCL1	Hs00236937_m1
C-C Motif Chemokine Ligand 2	CCL2	Hs00234140_m1
Interleukin 8	CXCL8	Hs00174103_m1
Angiopoietin-like 4	ANGPTL4	Hs00211522_m1
Stanniocalcin-1	STC1	Hs00174970_m1
E-cadherin	CDH1	Hs01023895_m1
Snail Family Transcriptional Repressor 1	Snail	Hs00195591_m1
3-Hydroxy-3-Methylglutaryl-CoA Reductase	HMGCR	Hs00168352_m1
Interleukin 6	IL6	Hs00985639_m1
C-X-C Motif Chemokine Ligand 12	CXCL12	Hs03676656_mH
Intercellular Adhesion Molecule 1	ICAM	Hs00164932_m1
Insulin Induced Gene 1	INSIG1	Hs01650977_g1
Fibronectin	FN1	Hs00365052_m1
Snail	Snai1	Hs00195591_m1
α-SMA	ACTA2	Hs00426835_g1
